# PIVKA-II and hematological parameters in hepatocellular carcinoma and non-malignant chronic liver disease: A case-control biomarker comparison in a hepatitis-endemic population

**DOI:** 10.1371/journal.pone.0361802

**Published:** 2026-11-03

**Authors:** Muhammad Sameer Hashmi, Muhammad Aqif Ilyas

**Affiliations:** 1 Department of Medical Laboratory Technology, Faculty of Natural Sciences, University of Sindh, Jamshoro, Sindh, Pakistan; 2 Department of Physiology, Faculty of Natural Sciences, University of Sindh, Jamshoro, Sindh, Pakistan; Fudan University, CHINA

## Abstract

**Background:**

Early detection of hepatocellular carcinoma (HCC) remains challenging in hepatitis-endemic settings such as Pakistan, where surveillance commonly relies on ultrasonography and alpha-fetoprotein (AFP), both of which have limited sensitivity for early-stage disease. This study evaluated the diagnostic performance of serum Protein Induced by Vitamin K Absence or Antagonist-II (PIVKA-II), complete blood count (CBC) parameters, and CBC-derived hematological indices for discriminating HCC from non-malignant chronic liver disease (CLD).

**Methods:**

We conducted a case-control biomarker study involving 160 adults with CLD at Liaquat University Hospital, Sindh, Pakistan, between October 2025 and March 2026, including 74 patients with established HCC and 86 with non-malignant CLD. Serum PIVKA-II was measured using electrochemiluminescence immunoassay. CBC-derived neutrophil-to-lymphocyte ratio, platelet-to-lymphocyte ratio, monocyte-to-lymphocyte ratio, systemic immune-inflammation index, and red cell distribution width-to-platelet ratio were calculated. Between-group differences were assessed using non-parametric methods, and receiver operating characteristic (ROC) analysis evaluated discriminatory performance. Benjamini–Hochberg correction was applied to prespecified families of multiple comparisons.

**Results:**

PIVKA-II demonstrated the strongest discriminatory performance, with an area under the ROC curve (AUC) of 0.753 (95% CI, 0.670–0.827). At a cutoff of >82.1 mAU/mL, sensitivity was 86.4% and specificity was 56.0%. Platelet count (AUC, 0.642) and the RDW-to-platelet ratio (AUC, 0.638) also demonstrated significant between-group differences that remained significant after false discovery rate correction, although their discriminatory performance was modest. PIVKA-II discrimination was substantially higher among patients with vascular invasion (AUC, 0.853) and advanced BCLC stage C/D disease (AUC, 0.853) than among those with early/intermediate BCLC A/B disease (AUC, 0.571). A combined model incorporating PIVKA-II, lymphocyte percentage, and platelet count yielded an AUC of 0.784, but did not significantly improve discrimination compared with PIVKA-II alone (p = 0.166). Bootstrap validation showed minimal optimism in the overall AUC; however, the optimal PIVKA-II cutoff demonstrated substantial variability, with a 95% percentile range of 58.7–715.8 mAU/mL. This was corroborated by an independent 10-fold cross-validation analysis, which yielded a comparable mean AUC of 0.751 for PIVKA-II (optimism = +0.002).

**Conclusion:**

PIVKA-II demonstrated fair diagnostic discrimination between HCC and non-malignant CLD, with relatively high sensitivity but limited specificity. Its substantially lower performance in early/intermediate-stage disease and the marked instability of the optimal cutoff indicate that PIVKA-II should be considered a complementary biomarker rather than a standalone diagnostic test. Larger multicenter studies with standardized control populations and independent external validation are needed to establish robust population-specific thresholds and determine the incremental clinical value of combining PIVKA-II with hematological parameters.

## Introduction

Hepatocellular carcinoma (HCC) is one of the most common malignant tumors worldwide and ranks third among the leading causes of cancer-related deaths, with more than 800,000 deaths annually [1]. Most of the burden falls on areas endemic to viral hepatitis, where chronic infections with HBV and HCV cause progressive liver damage, cirrhosis, and malignant transformation [2]. Pakistan’s prevalence of HBV and HCV is one of the highest globally, contributing to increased HCC incidence and placing a heavy burden on health systems [3, 4]. Beyond its high mortality, HCC also carries a substantial symptom burden, with cancer-related pain being one of the most significant factors diminishing patients’ quality of life and complicating clinical management [5]. In this regard, the most commonly used biomarkers, such as alpha-fetoprotein (AFP), have poor sensitivity in the early disease stage [6].

HCC develops in almost all cases of chronic liver disease (CLD), where 80–90% of HCC cases occur in patients with cirrhosis or long-term hepatic inflammation [1]. CLD is one of the strongest predictors of HCC development due to persistent liver injury, progressive fibrosis, architectural distortion, and ultimately malignant transformation [2]. In Pakistan, CLD is mainly attributable to hepatitis C and B viruses, accounting for most cases of cirrhosis and HCC in the country [3, 4]. Other causes, such as non-alcoholic fatty liver disease (NAFLD), alcohol abuse, and autoimmune liver disease, are increasingly recognised as significant contributors to chronic liver injury in this region [7].

Although early diagnosis of HCC offers the best opportunity to improve prognosis, it remains challenging because the tumour grows silently and conventional diagnostic tools have well-documented limitations. The current recommendations for confirming HCC include imaging, serum biomarkers, and clinical assessment. Triphasic contrast-enhanced CT and dynamic MRI are the diagnostic gold standards, owing to their characteristic pattern of arterial-phase hyper-enhancement with portal venous or delayed-phase washout [8]. However, small or atypical lesions may not display classic imaging features, making serum biomarkers such as AFP more relied upon. Despite its widespread use, AFP has limited sensitivity and specificity in the early stages of HCC and in the presence of active hepatitis or cirrhosis [6]. These limitations have driven interest in newer biomarkers such as Protein Induced by Vitamin K Absence or Antagonist-II (PIVKA-II), which demonstrates superior diagnostic accuracy and better discrimination of HCC from benign CLD [9].

Apart from tumour-specific biomarkers, inflammatory and hematological indices have also been investigated as potential adjuncts in HCC assessment. The neutrophil-to-lymphocyte ratio (NLR), platelet-to-lymphocyte ratio (PLR), and thrombocytopenia reflect the systemic inflammatory and hematological alterations associated with chronic liver disease and tumour progression. Elevated NLR and PLR have been more consistently associated with adverse tumour characteristics and clinical outcomes, including advanced tumour biology, poor differentiation, vascular invasion, and reduced survival, supporting their predominantly prognostic rather than diagnostic role in HCC [10–12]. Although these indices may provide complementary information when combined with established HCC biomarkers, their ability to independently discriminate HCC from non-malignant CLD remains less well established. Platelet abnormalities, particularly thrombocytopenia, are closely associated with the severity of cirrhosis and may provide additional information when evaluating patients with chronic liver disease at risk of HCC [13].

In Pakistan, HCC is a major complication of CLD, with viral hepatitis remaining highly prevalent, particularly in Sindh. Despite this burden, HCC diagnosis in this region still relies predominantly on ultrasonography and AFP, both of which frequently fail to detect early-stage disease. Growing evidence suggests that PIVKA-II, NLR, and hematological parameters such as platelet indices better discriminate HCC in high-risk patients; however, locally generated evidence on the combined diagnostic performance of PIVKA-II and CBC-derived inflammatory indices in this hepatitis-endemic population remains scarce. Reliable, accessible, and cost-effective diagnostic tools are urgently needed to enable earlier HCC detection in routine clinical practice.

The aim of this study was to evaluate the diagnostic performance of PIVKA-II levels, neutrophil-to-lymphocyte ratio, and platelet derangements in distinguishing hepatocellular carcinoma from non-HCC chronic liver disease in a viral hepatitis–endemic population of Sindh, Pakistan.

## Materials and methods

### Study design

A case-control biomarker study was conducted among 160 adults with CLD at Liaquat University Hospital, Sindh, Pakistan, between October 2025 and March 2026. Of these, 74 patients had established HCC based on histological or radiological findings, while 86 had non-malignant CLD. Serum PIVKA-II was measured using electrochemiluminescence immunoassay. CBC parameters were recorded, and the neutrophil-to-lymphocyte ratio (NLR), platelet-to-lymphocyte ratio (PLR), monocyte-to-lymphocyte ratio (MLR), systemic immune-inflammation index (SII), and RDW/platelet ratio were calculated. Groups were compared using non-parametric tests, and ROC analysis assessed how well these biomarkers distinguished established HCC from non-malignant CLD.

### Study setting

The study was carried out at Liaquat University Hospital (LUH), Hyderabad and Jamshoro campuses, major tertiary care teaching hospitals managing a high burden of liver disease across Sindh. All laboratory work, including CBC, peripheral blood smear preparation, and hepatitis serology, was performed at the Department of Physiology, Medical Laboratory Technology (MLT) and Public Health, University of Sindh, Jamshoro. Serum PIVKA-II analysis was conducted at the Diagnostic and Research Clinical Laboratory, Asian Institute of Medical Sciences (AIMS), Hyderabad, using electrochemiluminescence immunoassay (ECLIA).

### Study population

The study included adult patients aged 18 years and above with confirmed chronic liver disease of viral or non-viral etiology presenting to LUH during the study period. HCC diagnosis was established by the treating physician or gastroenterologist on the basis of non-invasive imaging criteria in accordance with the EASL 2018 Clinical Practice Guidelines: a hepatic nodule demonstrating characteristic arterial-phase hyperenhancement with portal venous or delayed-phase washout on triphasic contrast-enhanced CT or dynamic contrast-enhanced MRI was considered diagnostic without the need for histological confirmation, consistent with established non-invasive HCC diagnostic pathways [8]. In cases where imaging findings were atypical or inconclusive, histopathological confirmation was obtained. Non-malignant CLD was defined by the absence of any imaging or histological evidence of malignancy in patients with biopsy-proven or clinically established chronic liver disease. Only participants who had been

classified as HCC-positive or HCC-negative prior to enrolment were included. Participants were not required to bring outside laboratory reports; instead, all required CBC, smear, and serology tests were performed directly by the research team at the Department of Physiology and MLT as detailed below. Patients were excluded if they had other comorbidities known to significantly alter hematological or inflammatory markers, including hematological malignancies, active systemic infections such as sepsis or tuberculosis, chronic kidney disease (stage 3 or above), autoimmune disorders, HIV infection, uncontrolled diabetes mellitus, thyroid dysfunction, congestive heart failure, pregnancy, recent blood transfusion within four weeks, or any known extrahepatic malignancy. Patients with incomplete samples or missing laboratory data were also excluded.

### Sample collection and processing

Venous blood samples were collected by trained phlebotomists following standard aseptic procedures according to international guidelines for safe blood collection [14]. For in-patients, the CBC sample included in the study was the first CBC drawn at the time of hospital admission to ensure uniformity and prevent confounding from later disease progression or treatment. A 3 mL EDTA sample was obtained for CBC and peripheral smear preparation, and 5 mL of blood was collected in a plain tube for serum separation. EDTA samples were processed within two hours of collection. Serum samples were centrifuged, aliquoted, and stored at −20°C until analysis in accordance with recommended laboratory specimen handling procedures [15]. Aliquots intended for PIVKA-II testing were transported to AIMS Hyderabad under cold-chain conditions.

### Laboratory analysis

CBC with five-part differential was performed using an automated hematology analyzer (XN-550, Sysmex Corporation, Kobe, Japan), providing hemoglobin, red cell indices, leukocyte differential counts, and platelet count, following standard hematology laboratory protocols [16]. Internal quality control was performed using commercially available Sysmex XN CHECK hematology control material at appropriate concentration levels, with control results monitored against manufacturer-provided target values and acceptable ranges before and during routine sample analysis, and quality-control data maintained within the analyzer’s quality-control system. External analytical quality was assessed through inter-laboratory comparison: selected patient specimens were analyzed independently in the study laboratory and a collaborating laboratory, and results were compared to evaluate analytical agreement and consistency of CBC measurements. Peripheral blood smears were stained with Leishman stain and examined under oil immersion for red cell morphology, white cell distribution, and platelet adequacy, according to established microscopic examination guidelines [17]. Hepatitis serology, including Anti-HCV and HBsAg, was performed using ELISA and reported as signal-to-cutoff ratios in line with World Health Organization recommendations for viral hepatitis testing [18].

### PIVKA-II quantification

Serum PIVKA-II levels were measured using an electrochemiluminescence immunoassay (ECLIA) on the Roche Cobas e 411 analyzer at AIMS Hyderabad. The assay used in this study was from lot number **10 08273001**. PIVKA-II was quantitatively measured according to the manufacturer’s instructions and has been widely used as a biomarker for hepatocellular carcinoma [19]. The analytical measurement range of the assay was **6.76–12,000 mAU/mL**, with concentrations below **40 mAU/mL** considered within the normal range according to the manufacturer’s validation data [20]. Samples with concentrations exceeding the upper analytical measurement limit were recorded as **≥12,000 mAU/mL** for statistical analysis.

For analytical quality assurance, **inter-laboratory verification** was performed using reference/quality-control samples provided through an external laboratory. The same samples were analyzed independently, and the PIVKA-II results obtained at AIMS Hyderabad were compared with the corresponding results reported by the reference laboratory. The observed inter-laboratory results were reviewed for agreement to verify the analytical performance and consistency of the PIVKA-II assay during the study period.

### Derived hematological indices

Three inflammatory indices were calculated from CBC parameters: neutrophil-to-lymphocyte ratio (NLR), platelet-to-lymphocyte ratio (PLR), and monocyte-to-lymphocyte ratio (MLR). These ratios were used to assess systemic inflammatory and immunological alterations associated with HCC. Additional derived indices calculated included the lymphocyte-to-monocyte ratio (LMR), the systemic immune-inflammation index (SII = neutrophils × platelets/ lymphocytes), and the RDW-to-platelet ratio (RDW/PLT = RDW-CV%/ platelet count in ×10^9^/L × 100). All indices were calculated from the same CBC specimen.

### Data collection and statistical analysis

Data were entered into a structured Excel file and verified for accuracy. Statistical analysis was performed using IBM SPSS Statistics version 29.0 (Standard Edition; IBM Corp., Armonk, NY, USA). Continuous variables were summarized as mean ± standard deviation or median with interquartile range (IQR), while categorical variables were expressed as frequencies and percentages. Because the majority of continuous parameters failed the Shapiro–Wilk normality test, non-parametric methods were used throughout: the Mann–Whitney U test for between-group comparisons and Spearman’s rank correlation for association analyses. Chi-square tests were applied for categorical comparisons. The sample size was based on consecutive eligible patients presenting during the six-month study period; no formal a priori power calculation was performed, and all available cases meeting inclusion criteria were enrolled. Receiver operating characteristic (ROC) curve analysis was performed to assess the discriminatory ability of each marker. Optimal cut-off values were selected by maximising the Youden index (sensitivity + specificity − 1). Area under the ROC curve (AUC) values are reported with 95% bootstrap confidence intervals (1,000 iterations). Sensitivity, specificity, positive predictive value (PPV), and negative predictive value (NPV) are reported at the optimal cut-off. To further assess the internal validity of the ROC-derived cutoffs, stratified 10-fold cross-validation (10 splits × 50 repeats = 500 train/test iterations; Python 3.12, scikit-learn v1.8) was additionally performed for each marker. In each iteration, the Youden-optimal cutoff and classification direction were determined using only the training folds (90% of the data) and applied, without re-tuning, to the corresponding held-out 10% test fold to obtain out-of-sample AUC, sensitivity, and specificity. The mean, standard deviation, and 2.5th–97.5th percentile range of the 500 held-out test-fold AUCs are reported as the cross-validated AUC. Optimism was calculated as apparent AUC minus mean cross-validated AUC, analogous to the bootstrap optimism described above; this provided an independent, non-resampling-based check on the bootstrap internal validation. Effect size for Mann–Whitney U comparisons was expressed as the rank-biserial correlation coefficient r, interpreted as negligible (|r| < 0.10), small (0.10–0.29), medium (0.30–0.49), or large (≥ 0.50). A p-value less than 0.05 was considered statistically significant. Given the number of CBC parameters and derived hematological indices compared, corrections for multiple testing were applied within each family of comparisons (routine CBC parameters and PIVKA-II, Table 5; derived hematological ratios, Table 6; PIVKA-II correlations, Table 7) using the Benjamini–Hochberg procedure to control the false discovery rate (FDR) at q < 0.05. FDR control, rather than the more conservative Bonferroni correction, was chosen because CBC-derived parameters and ratios are not statistically independent (several ratios share component cell counts), and Bonferroni correction is known to be overly conservative under such correlation structures. Both nominal (unadjusted) and FDR-adjusted p-values are reported below and in Tables 5–7; findings significant only at the nominal level are interpreted as exploratory/hypothesis-generating rather than confirmatory.

### Ethical considerations

Ethical approval was obtained from the Institutional Review Board, Department of Physiology, Faculty of Natural Sciences, University of Sindh, Jamshoro (Approval reference: Physio/IRB/235). All procedures adhered to the principles of the Declaration of Helsinki for research involving human participants [21]. Written informed consent was obtained from every participant in accordance with IRB-approved consent procedures, and patient confidentiality was ensured through anonymized data handling and secure record management. Because the study data contain human participant information, access to the individual-level dataset is subject to patient confidentiality, privacy, and institutional data-governance requirements. The conditions and procedures for accessing de-identified data are detailed in the Data Availability Statement.

## Results

### Study population and baseline characteristics

A total of 160 patients were included in the final analysis, comprising 74 patients (46.3%) with hepatocellular carcinoma (HCC) and 86 patients (53.7%) with non-malignant chronic liver disease (CLD). The distribution of HCC and non-malignant CLD patients is illustrated in Fig 1A, while hepatitis serology profiles by study group are presented in Fig 1B. Baseline demographic and clinical characteristics are summarized in Table 1.

**Table 1:** Baseline demographic and clinical characteristics.

*Variable*	*Total (n = 160)*	*HCC (n = 74)*	*Non-Malignant CLD (n = 86)*	*p-value*
*Total Patients, n (%)*	*160*	*74 (46.3%)*	*86 (53.7%)*	*—*
*Age (years), Mean ± SD*	*55.4 ± 10.7*	*54.9 ± 10.6*	*55.9 ± 10.9*	*0.233*
*Age, Median (IQR)*	*56 (48–64)*	*56 (47–63)*	*57 (48–64)*	
***Gender, n (%)***				***0.461***
*Male*	*100 (62.5%)*	*49 (66.2%)*	*51 (59.3%)*	
*Female*	*60 (37.5%)*	*25 (33.8%)*	*35 (40.7%)*	
*Anti-HCV Reactive, n (%)*	*133 (83.1%)*	*67 (90.5%)*	*66 (76.7%)*	0.020*
*HBsAg Reactive, n (%)*	*20 (12.5%)*	*7 (9.5%)*	*13 (15.1%)*	*0.281*
*Hepatitis Delta Ab, n (%)*	*1 (0.6%)*	*0 (0.0%)*	*1 (1.2%)*	*—*
***Cirrhosis Status, n (%)***				***<0.001***
*No cirrhosis*	*33 (20.6%)*	*7 (9.5%)*	*26 (30.2%)*	
*Compensated cirrhosis*	*48 (30.0%)*	*15 (20.3%)*	*33 (38.4%)*	
*Decompensated cirrhosis*	*79 (49.4%)*	*52 (70.3%)*	*27 (31.4%)*	

The mean age of the overall cohort was 55.4 ± 10.7 years, with no significant difference between the HCC and non-malignant CLD groups (54.9 ± 10.6 vs. 55.9 ± 10.9 years, respectively; p = 0.233). The study population was predominantly male (62.5%), with a comparable sex distribution between groups (66.2% vs. 59.3% male in the HCC and CLD groups, respectively; p = 0.461). Anti-HCV reactivity was detected in 133 patients (83.1%) overall and was significantly more frequent among HCC patients than non-malignant CLD controls (90.5% vs. 76.7%; p = 0.020). HBsAg reactivity was detected in 20 patients (12.5%), with no significant difference between groups (9.5% vs. 15.1%; p = 0.281). Dual negative serology was observed in 2 patients (2.7%) in the HCC group and 11 patients (12.8%) in the CLD group, while one patient (0.6%) overall was positive for hepatitis delta antibody and belonged to the non-malignant CLD group.

Cirrhosis status differed significantly between the two groups (p < 0.001). Decompensated cirrhosis was present in 52 (70.3%) HCC patients compared with 27 (31.4%) non-malignant CLD controls, whereas compensated cirrhosis was observed in 15 (20.3%) and 33 (38.4%) patients, respectively. No cirrhosis was present in 7 (9.5%) HCC patients and 26 (30.2%) CLD controls. These findings demonstrate a greater burden of advanced underlying liver disease among patients with HCC.

**Fig 1:**
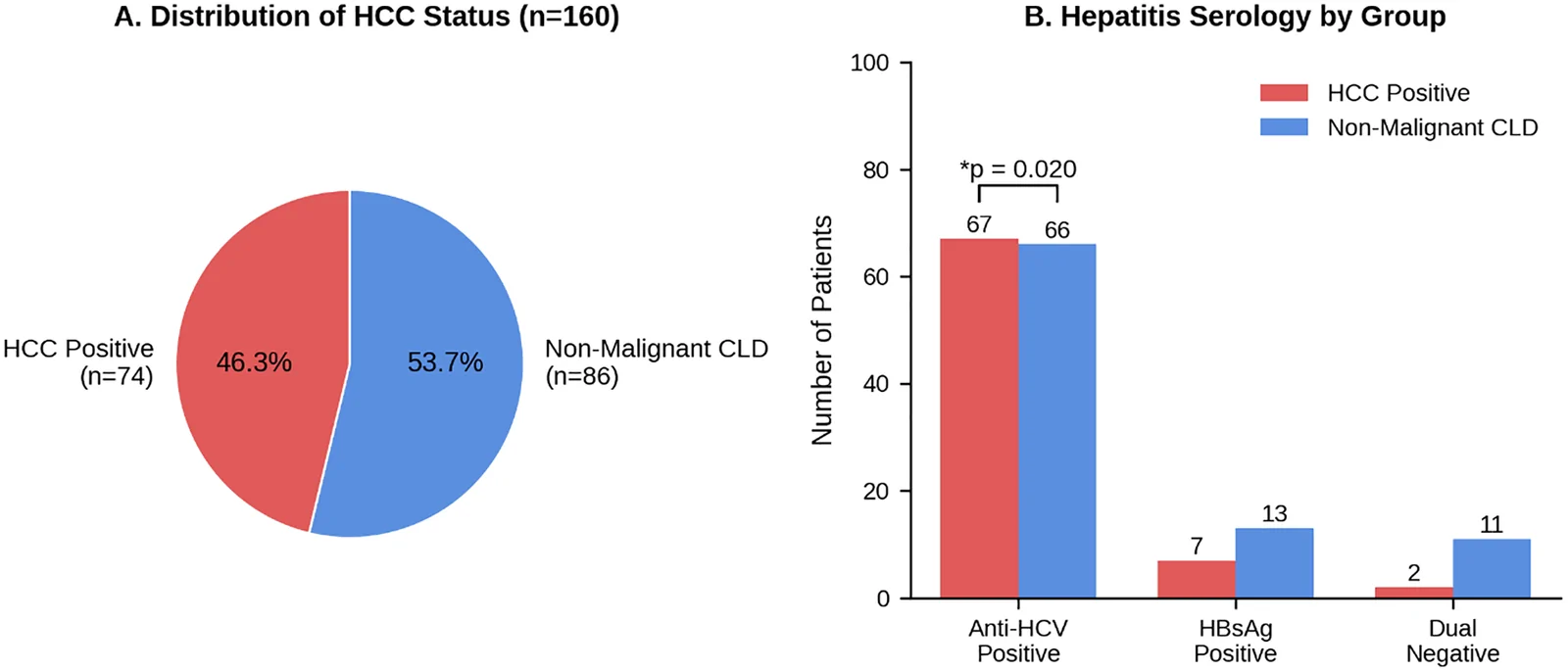
(A) Distribution of HCC vs. non-malignant CLD in the study population (n = 160). (B) Hepatitis serology profiles by group. *p = 0.020 for Anti-HCV reactivity (Chi-square test).

### Tumor characteristics, BCLC stage, and treatment status

Detailed tumor characteristics of the 74 HCC patients are presented in Table 2. The mean tumor size was 6.16 ± 2.62 cm, with a median of 6.15 cm (IQR, 4.03–8.00 cm). Tumors >5 cm were present in 45 patients (60.8%), whereas 29 patients (39.2%) had tumors ≤5 cm. The median number of lesions was 3 (IQR, 2–4), with multiple lesions observed in 66 patients (89.2%) and a single lesion in 8 patients (10.8%). Vascular invasion was present in 43 patients (58.1%) and absent in 31 (41.9%). Regarding BCLC stage, 4 patients (5.4%) were classified as stage A, 22 (29.7%) as stage B, 33 (44.6%) as stage C, and 15 (20.3%) as stage D. Overall, 48 patients (64.9%) had advanced-stage disease (BCLC C/D), whereas 26 (35.1%) had early/intermediate-stage disease (BCLC A/B). Cirrhosis was present in 67 patients (90.5%), including 15 (20.3%) with compensated cirrhosis and 52 (70.3%) with decompensated cirrhosis. In this study, “early-stage HCC” was defined strictly according to the Barcelona Clinic Liver Cancer (BCLC) staging system as BCLC stage A (single tumor of any size, or up to three nodules each ≤3 cm, without vascular invasion or extrahepatic spread, in patients with preserved liver function and performance status), whereas BCLC stage B was classified as intermediate-stage disease. By this strict definition, only 4 patients (5.4%) in the present cohort had early-stage (BCLC-A) HCC; the larger early/intermediate group (BCLC A + B, n = 26, 35.1%) reported above and used in subgroup analyses therefore combines early-stage and intermediate-stage patients and should not be equated with early-stage disease alone. Given the very small number of strictly early-stage cases, biomarker performance estimates specific to early-stage HCC could not be reliably derived from this cohort.

**Table 2:** Tumor Burden, BCLC Stage, cirrhosis status, and treatment characteristics among newly diagnosed HCC patients (n = 74).

*Characteristic*	*HCC Patients (n = 74)*
***Tumor size (cm)***	
*Mean ± SD*	*6.16 ± 2.62*
*Median (IQR)*	*6.15 (4.03–8.00)*
*≤ 5 cm, n (%)*	*29 (39.2%)*
*> 5 cm, n (%)*	*45 (60.8%)*
***Number of lesions***	
*Median (IQR)*	*3 (2–4)*
*Single lesion, n (%)*	*8 (10.8%)*
*Multiple lesions (>1), n (%)*	*66 (89.2%)*
***Vascular invasion, n (%)***	
*Present*	*43 (58.1%)*
*Absent*	*31 (41.9%)*
***BCLC stage, n (%)***	
*A*	*4 (5.4%)*
*B*	*22 (29.7%)*
*C*	*33 (44.6%)*
*D*	*15 (20.3%)*
***Underlying cirrhosis, n (%)***	
*No cirrhosis*	*7 (9.5%)*
*Compensated*	*15 (20.3%)*
*Decompensated*	*52 (70.3%)*
***HCC diagnosis status, n (%)***	
*Newly diagnosed HCC*	***74 (100%)***
*Previously diagnosed HCC*	***0 (0%)***
***Anticancer treatment at enrollment, n (%)***	
*Not receiving anticancer treatment*	***74 (100%)***
*Receiving anticancer treatment*	***0 (0%)***

All 74 HCC patients were newly diagnosed at enrollment, and none had previously received HCC-directed anticancer treatment. Therefore, all biomarker and hematological measurements were obtained before initiation of anticancer therapy.

### Descriptive statistics of CBC parameters and PIVKA-II

Descriptive statistics for CBC parameters and PIVKA-II are presented in Table 3, with median CBC values illustrated in Fig 2. PIVKA-II levels were markedly higher in HCC patients than in non-malignant CLD controls. The mean PIVKA-II concentration was 576.2 ± 710.2 mAU/mL in HCC patients compared with 110.1 ± 197.4 mAU/mL in the CLD group, while the corresponding median values were 461.6 (IQR, 151–1186) and 59.6 (IQR, 30–115) mAU/mL, respectively.

**Table 3:** Descriptive statistics of CBC parameters and PIVKA-II by group.

*Parameter*	*Ref. Range*	*HCC (n = 74)*		*Non-Malignant CLD (n = 86)*	
		*Mean ± SD*	*Median (IQR)*	*Mean ± SD*	*Median (IQR)*
*Hemoglobin (g/dL)*	*11.3–17.3*	*10.46 ± 2.87*	*9.9 (8.2–12.6)*	*9.72 ± 2.86*	*9.9 (7.8–11.5)*
*RBCs (×10^12^/L)*	*4.5–5.5*	*4.00 ± 0.72*	*4.01 (3.5–4.5)*	*3.94 ± 0.64*	*3.9 (3.4–4.5)*
*Hematocrit (%)*	*40–52*	*32.4 ± 8.3*	*32.0 (27–37)*	*32.3 ± 7.9*	*32.2 (27–37)*
*MCV (fL)*	*80–100*	*84.7 ± 13.2*	*84.7 (77–91)*	*83.5 ± 9.5*	*82.8 (79–90)*
*MCH (pg)*	*27–33*	*27.2 ± 4.6*	*27.2 (24–30)*	*27.4 ± 3.7*	*27.1 (25–30*)
*MCHC (g/dL)*	*32–36*	*32.2 ± 2.3*	*32.4 (31–34)*	*32.7 ± 2.1*	*32.4 (31–34)*
*RDW-CV (%)*	*11.5–14.5*	*17.9 ± 3.3*	*17.55 (16–20)*	*16.5 ± 2.9*	*16.75 (15–18)*
*TLC (×10^9^/L)*	*4.0–11.0*	*6.75 ± 2.68*	*6.48 (5.1–8.4)*	*6.77 ± 2.34*	*6.60 (5.3–8.1)*
*Neutrophils (%)*	*40–75*	*69.2 ± 11.1*	*70.7 (62–78)*	*65.3 ± 11.0*	*64.8 (58–73)*
*Lymphocytes (%)*	*20–45*	*18.2 ± 9.4*	*17.35 (12–23)*	*20.5 ± 9.0*	*20.5 (15–27)*
*Monocytes (%)*	*2–10*	*8.9 ± 3.1*	*8.5 (7–10)*	*8.8 ± 2.7*	*8.9 (7–10)*
*Eosinophils (%)*	*1–4*	*2.3 ± 1.5*	*2.05 (1–3)*	*2.3 ± 1.5*	*2.2 (1–3)*
*Basophils (%)*	*0–1*	*0.37 ± 0.26*	*0.3 (0.2–0.5)*	*0.37 ± 0.28*	*0.3 (0.2–0.5)*
*Platelets (×10^9^/L)*	*150–400*	*120.4 ± 88.3*	*110 (68–161)*	*152.1 ± 87.0*	*150 (100–188)*
*PIVKA-II (mAU/mL)*	*< 40*	*576.2 ± 710.2*	*461.6 (151–* *1186)*	*110.1 ± 197.4*	*59.6 (30–115)*

Several hematological parameters showed clinically relevant differences between groups. Median platelet count was lower among HCC patients than CLD controls (110 vs. 150 × 10^9^/L). Median neutrophil percentage was higher in HCC patients (70.7% vs. 64.8%), whereas median lymphocyte percentage was lower (17.35% vs. 20.5%). Median RDW-CV was also numerically higher in HCC patients (17.55% vs. 16.75%). Other CBC parameters showed broadly comparable distributions between groups.

**Fig 2:**
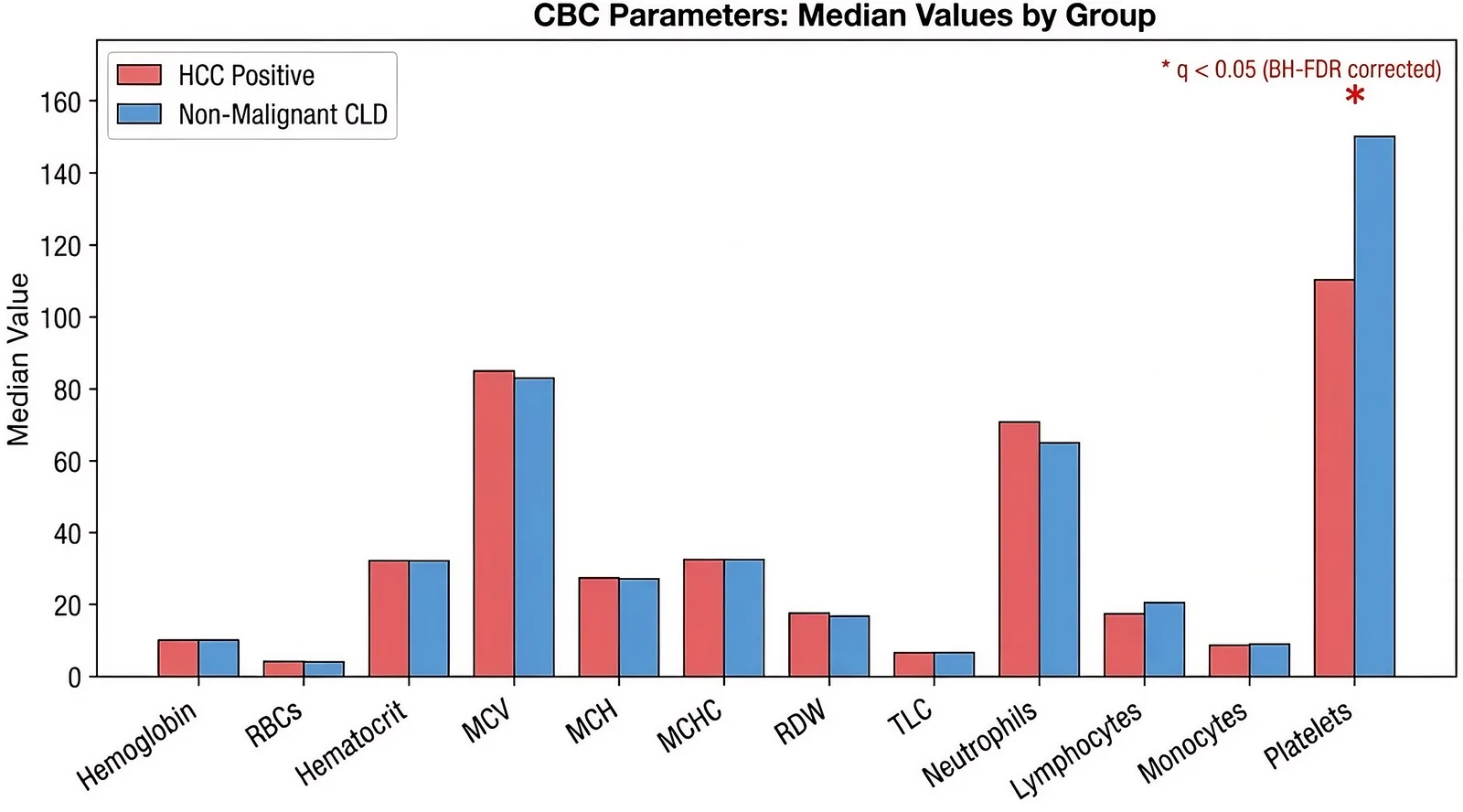
Grouped bar chart showing median CBC values for HCC vs. non-malignant CLD. Asterisk (*) denotes p < 0.05 (Mann-Whitney U test).

### Normality assessment

The Shapiro–Wilk test was used to assess the distribution of continuous variables (Table 4). Several parameters demonstrated significant deviation from normality in at least one study group, particularly RDW-CV, TLC, lymphocyte percentage, monocyte percentage, eosinophil percentage, basophil percentage, platelet count, PIVKA-II, and all derived hematological ratios. PIVKA-II and all evaluated hematological ratios showed non-normal distributions in both groups (p < 0.001). Accordingly, non-parametric statistical methods were used for between-group comparisons and correlation analyses.

**Table 4:** Shapiro-wilk normality test results for all parameters.

*Parameter*	*HCC (n = 74)*			*Non-Malignant CLD (n = 86)*			*Overall*
	*W*	*p*	*Distribution*	*W*	*p*	*Distribution*	
*Hemoglobin (g/dL)*	*0.9836*	*0.341*	*Normal*	*0.9907*	*0.379*	*Normal*	*Normal*
*RBCs (×10^12^/L)*	*0.9812*	*0.282*	*Normal*	*0.9831*	*0.049*	***Non-Normal***	*—*
*Hematocrit (%)*	*0.9864*	*0.472*	*Normal*	*0.9926*	*0.587*	*Normal*	*Normal*
*MCV (fL)*	*0.9821*	*0.303*	*Normal*	*0.9851*	*0.083*	*Normal*	*Normal*
*MCH (pg)*	*0.9778*	*0.211*	*Normal*	*0.9791*	*0.016*	***Non-Normal***	*—*
*MCHC (g/dL)*	*0.9753*	*0.162*	*Normal*	*0.9795*	*0.018*	***Non-Normal***	*—*
*RDW-CV (%)*	*0.9264*	*<0.001*	***Non-Normal***	*0.9472*	*<0.001*	***Non-Normal***	***Non-Normal***
*TLC (×10^9^/L)*	*0.7891*	*<0.001*	***Non-Normal***	*0.8044*	*<0.001*	***Non-Normal***	***Non-Normal***
*Neutrophils (%)*	*0.9814*	*0.289*	*Normal*	*0.9857*	*0.099*	*Normal*	*Normal*
*Lymphocytes (%)*	*0.9635*	*0.027*	***Non-Normal***	*0.9712*	*0.002*	***Non-Normal***	***Non-Normal***
*Monocytes (%)*	*0.9502*	*0.005*	***Non-Normal***	*0.9703*	*0.002*	***Non-Normal***	***Non-Normal***
*Eosinophils (%)*	*0.8173*	*<0.001*	***Non-Normal***	*0.8433*	*<0.001*	***Non-Normal***	***Non-Normal***
*Basophils (%)*	*0.8781*	*<0.001*	***Non-Normal***	*0.8834*	*<0.001*	***Non-Normal***	***Non-Normal***
*Platelets (×10^9^/L)*	*0.8412*	*<0.001*	***Non-Normal***	*0.8645*	*<0.001*	***Non-Normal***	***Non-Normal***
*PIVKA-II* *(mAU/mL)*	*0.4581*	*<0.001*	***Non-Normal***	*0.4883*	*<0.001*	***Non-Normal***	***Non-Normal***
*NLR*	*0.6401*	*<0.001*	***Non-Normal***	*0.6609*	*<0.001*	***Non-Normal***	***Non-Normal***
*PLR*	*0.5122*	*<0.001*	***Non-Normal***	*0.5331*	*<0.001*	***Non-Normal***	***Non-Normal***
*MLR*	*0.7862*	*<0.001*	***Non-Normal***	*0.8042*	*<0.001*	***Non-Normal***	***Non-Normal***
*LMR*	*0.5991*	*<0.001*	***Non-Normal***	*0.6200*	*<0.001*	***Non-Normal***	***Non-Normal***
*SII*	*0.5011*	*<0.001*	***Non-Normal***	*0.5169*	*<0.001*	***Non-Normal***	***Non-Normal***
*RDW/PLT Ratio*	*0.7091*	*<0.001*	***Non-Normal***	*0.7229*	*<0.001*	***Non-Normal***	***Non-Normal***

### Between-group comparisons of CBC parameters, PIVKA-II, and ratios

Mann–Whitney U test results for CBC parameters and hematological ratios are presented in Tables 5 and 6, with median ratio values illustrated in Fig 3 and PIVKA-II distributions shown in Fig 4. PIVKA-II demonstrated the largest between-group difference, with median concentrations of 461.6 mAU/mL in HCC patients and 59.6 mAU/mL in CLD controls (U = 4172.5, p < 0.001; r = 0.505). Platelet counts were significantly lower in HCC patients (110 vs. 150 × 10^9^/L; U = 2216.0, p = 0.004; r = 0.269). Neutrophil percentages were higher (70.7% vs. 64.8%; U = 3792.0, p = 0.037; r = 0.192), whereas lymphocyte percentages were lower (17.35% vs. 20.5%; U = 2582.0, p = 0.040; r = 0.189).

**Table 5:** Mann-Whitney U Test — CBC Parameters and PIVKA-II (HCC vs. Non-Malignant CLD).

*Parameter*	*HCC* *Median (IQR)*	*CLD* *Median (IQR)*	*U*	*p-value*	*q-value* *(BH-FDR)*	*Effect r*	*Magnitude*	*Sig.*
*Hemoglobin (g/dL)*	*10.35 (8.2–* *12.6)*	*9.90 (7.8–* *11.5)*	*3047.0*	*0.645*	*0.794*	*0.042*	*Small*	*NS*
*RBCs (×10^12^/L)*	*3.97 (3.5–* *4.5)*	*4.07 (3.4–* *4.5)*	*2913.5*	*0.359*	*0.754*	*0.084*	*Small*	*NS*
*Hematocrit (%)*	*32.0 (27–37)*	*32.2 (27–37)*	*3059.0*	*0.675*	*0.794*	*0.039*	*Small*	*NS*
*MCV (fL)*	*84.7 (77–91)*	*82.8 (79–90)*	*3388.5*	*0.481*	*0.777*	*0.065*	*Small*	*NS*
*MCH (pg)*	*27.2 (24–30)*	*27.1 (25–30)*	*3288.0*	*0.718*	*0.794*	*0.033*	*Small*	*NS*
*MCHC (g/dL)*	*32.4 (31–34)*	*32.4 (31–34)*	*3033.5*	*0.612*	*0.794*	*0.047*	*Small*	*NS*
*RDW-CV (%)*	*17.55 (16–20)*	*16.75 (15–18)*	*3752.0*	*0.051*	*0.153*	*0.179*	*Small*	*NS*
*TLC (×10^9^/L)*	*6.48 (5.1–* *8.4)*	*6.60 (5.3–* *8.1)*	*3117.5*	*0.827*	*0.831*	*0.020*	*Negligible*	*NS*
*Neutrophils (%)*	*70.7 (62–78)*	*64.8 (58–73)*	*3792.0*	0.037*	*0.143 (NS)*	***0.192***	*Small*	*NS*
*Lymphocytes (%)*	*17.35 (12–23)*	*20.5 (15–27)*	*2582.0*	0.040*	*0.143 (NS)*	***0.189***	*Small*	*NS*
*Monocytes (%)*	*8.5 (7–10)*	*8.9 (7–10)*	*3064.5*	*0.689*	*0.794*	*0.037*	*Small*	*NS*
*Eosinophils (%)*	*2.05 (1–3)*	*2.20 (1–3)*	*2954.0*	*0.436*	*0.763*	*0.072*	*Small*	*NS*
*Basophils (%)*	*0.30 (0.2–* *0.5)*	*0.30 (0.2–* *0.5)*	*2941.0*	*0.407*	*0.763*	*0.076*	*Small*	*NS*
*Platelets (×10^9^/L)*	*110 (68–161)*	*150 (100–* *188)*	*2216.0*	0.004**	***0.028***	***0.269***	*Small*	*
*PIVKA-II* *(mAU/mL)*	*461.6 (151–* *1186)*	*59.6 (30–* *115)*	*4172.5*	<0.001***	***0.011***	***0.505***	*Medium*	*

**Table 6:** Hematological ratios — mann-whitney U test (HCC vs. Non-Malignant CLD).

Ratio	HCC Median (IQR)	CLD Median (IQR)	U	p-value	q-value (BH-FDR)	Effect r	Magnitude	Sig.
NLR	4.04 (2.9–6.1)	3.04 (2.1–4.6)	3780.0	0.041*	0.143 (NS)	**0.188**	Small	NS
PLR	6.92 (4.0–10.8)	6.73 (4.1–10.3)	3090.0	0.831	0.831	0.020	Negligible	NS
MLR	0.52 (0.35–0.77)	0.43 (0.28–0.62)	3704.0	0.074	0.173	0.164	Small	NS
LMR	1.92 (1.30–2.88)	2.32 (1.61–3.55)	2660.0	0.074	0.173	0.164	Small	NS
SII	451.3 (214–831)	415.0 (198–773)	3210.0	0.523	0.784	0.060	Negligible	NS
RDW/PLT Ratio	0.154 (0.09– 0.26)	0.116 (0.07– 0.18)	3868.5	0.003**	**0.028**	**0.277**	Small	*

Among the derived hematological ratios, NLR was higher in HCC patients than in non-malignant CLD controls (median, 4.04 [IQR, 2.9–6.1] vs. 3.04 [IQR, 2.1–4.6]; U = 3780.0, p = 0.041; r = 0.188). The RDW-to-platelet ratio was also higher in HCC patients (0.154 vs. 0.116; U = 3868.5, p = 0.003; r = 0.277). MLR and LMR showed non-significant trends (both p = 0.074), whereas PLR and SII did not differ significantly between groups.

**Fig 3:**
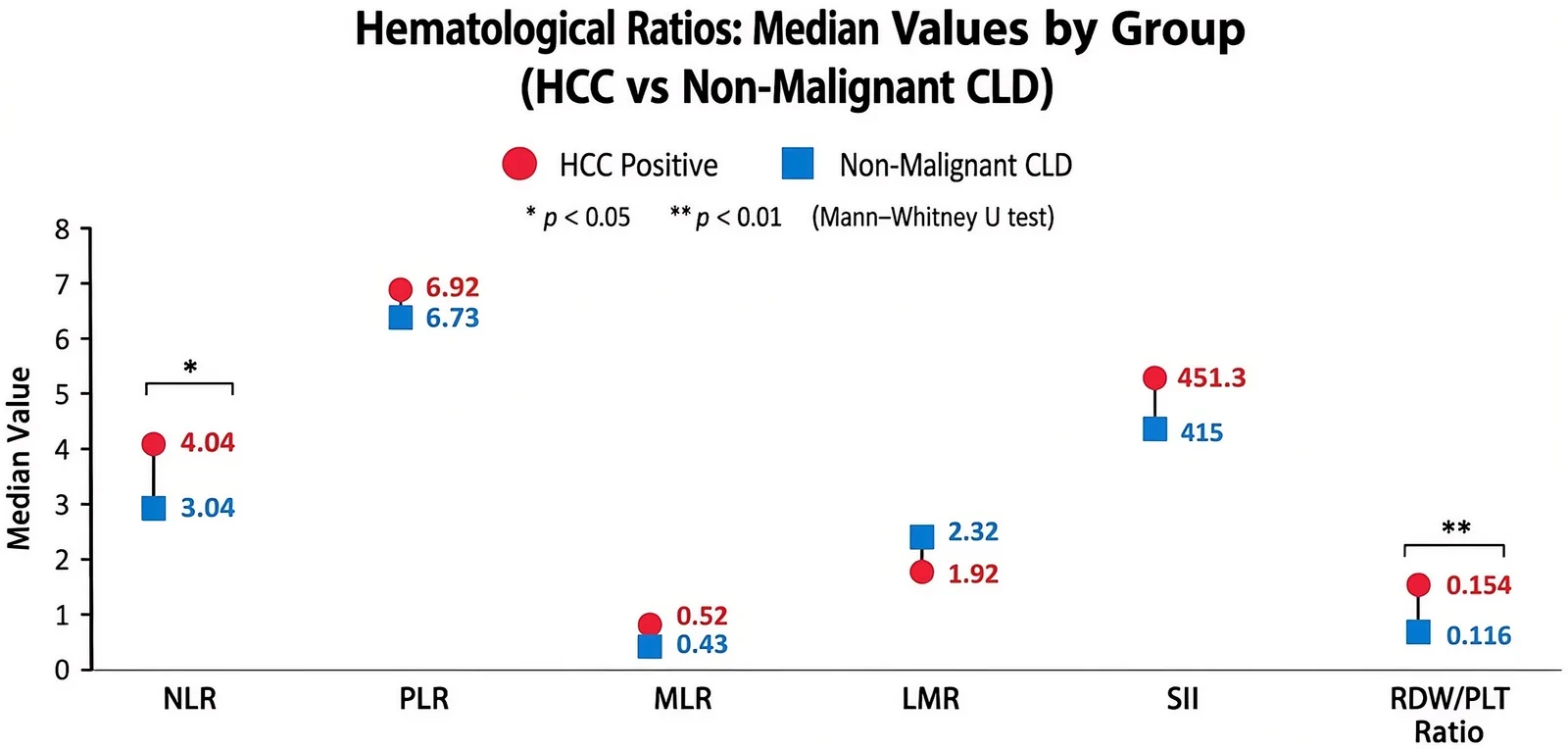
Lollipop chart of median hematological ratios for HCC vs. non-malignant CLD. *p < 0.05; **p < 0.01 (Mann-Whitney U test).

**Fig 4:**
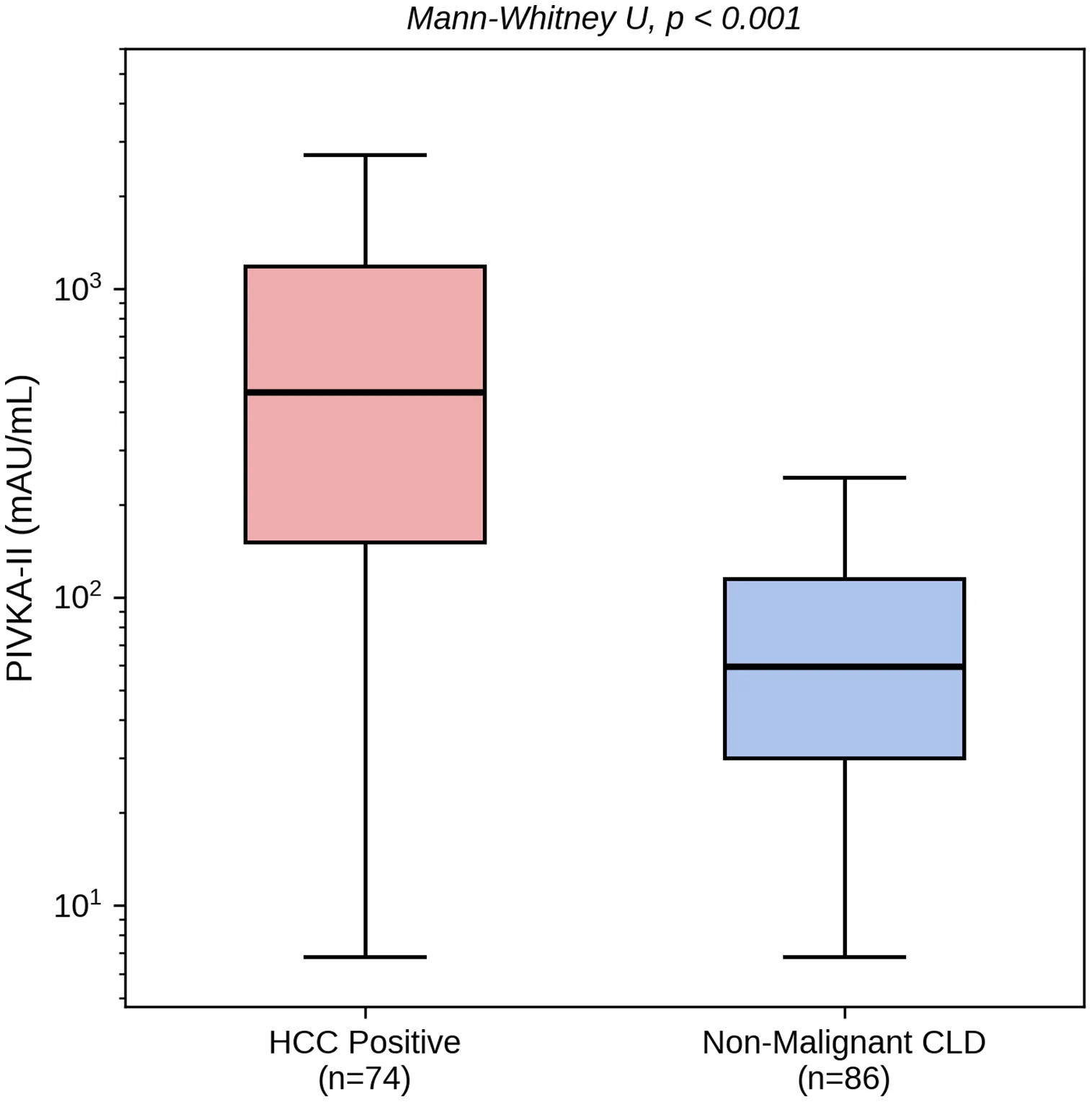
Box-and-whisker plot (log scale) of PIVKA-II levels in HCC vs. non-malignant CLD. Median: HCC = 461.6 mAU/mL; CLD = 59.6 mAU/mL. Mann-Whitney U p < 0.001.

To account for multiple testing, Benjamini–Hochberg FDR correction was applied across the 21 between-group comparisons represented in Tables 5 and 6. After correction, PIVKA-II (q = 0.011), platelet count (q = 0.028), and the RDW-to-platelet ratio (q = 0.028) remained statistically significant. In contrast, neutrophil percentage (q = 0.143), lymphocyte percentage (q = 0.143), and NLR (q = 0.143) did not survive FDR correction and were therefore considered exploratory findings. No statistically significant differences were observed for hemoglobin, RBC count, hematocrit, MCV, MCH, MCHC, RDW-CV, TLC, monocyte percentage, eosinophil percentage, or basophil percentage after correction.

### Correlation analysis of PIVKA-II with hematological parameters and tumor characteristics

Spearman rank correlation analyses are presented in Table 7 and illustrated in Fig 5. Across the full cohort (n = 160), PIVKA-II showed significant positive correlations with RDW-CV (ρ = 0.2326, p = 0.0031), monocyte percentage (ρ = 0.1693, p = 0.0324), and MLR (ρ = 0.2407, p = 0.0022). No significant correlations were observed between PIVKA-II and hemoglobin, RBC count, hematocrit, MCV, MCH, MCHC, TLC, neutrophil percentage, lymphocyte percentage, eosinophil percentage, basophil percentage, platelet count, NLR, PLR, LMR, ELR, BLR, or SII.

**Table 7:** Spearman rank correlation — PIVKA-II vs. CBC parameters and hematological ratios.

*Parameter*	*n*	*Spearman ρ*	*p-value*	*q-value (BH-FDR)*	*Significance*
*Hemoglobin (g/dL)*	*160*	*-0.0231*	*0.7715*	*0.857*	*NS*
*RBCs (×10^12^/L)*	*160*	*-0.0334*	*0.6747*	*0.844*	*NS*
*Hematocrit (%)*	*160*	*-0.0298*	*0.7086*	*0.844*	*NS*
*MCV (fL)*	*160*	*-0.0145*	*0.8553*	*0.900*	*NS*
*MCH (pg)*	*160*	*-0.0302*	*0.7047*	*0.844*	*NS*
*MCHC (g/dL)*	*160*	*-0.0289*	*0.7171*	*0.844*	*NS*
*RDW-CV (%)*	*160*	*0.2326*	*0.0031*	***0.031***	*
*TLC (×10^9^L)*	*160*	*0.0867*	*0.2759*	*0.521*	*NS*
*Neutrophils (%)*	*160*	*0.1323*	*0.0954*	*0.273*	*NS*
*Lymphocytes (%)*	*160*	*-0.1491*	*0.0598*	*0.223*	*NS*
*Monocytes (%)*	*160*	*0.1693*	*0.0324*	*0.216 (NS)*	*NS*
*Eosinophils (%)*	*160*	*-0.1452*	*0.0670*	*0.223*	*NS*
*Basophils (%)*	*160*	*-0.0717*	*0.3674*	*0.565*	*NS*
*Platelets (×10^9^/L)*	*160*	*-0.1082*	*0.1732*	*0.414*	*NS*
*NLR*	*160*	*0.1464*	*0.0647*	*0.223*	*NS*
*PLR*	*160*	*0.0803*	*0.3127*	*0.521*	*NS*
*MLR*	*160*	*0.2407*	*0.0022*	***0.031***	*
*ELR*	*160*	*-0.0803*	*0.3126*	*0.521*	*NS*
*BLR*	*160*	*0.0062*	*0.9376*	*0.938*	*NS*
*SII*	*160*	*0.1051*	*0.1861*	*0.414*	*NS*
*Tumor size (cm)*	*74*	*0.3210*	*0.0053*	***0.008***	**
*No. of lesions*	*74*	*-0.0020*	*0.9854*	*0.985*	*NS*
*Vascular invasion*	*74*	*0.6330*	*<0.0001*	***0.0002***	***

Among HCC patients (n = 74), PIVKA-II demonstrated a significant positive correlation with tumor size (ρ = 0.3210, p = 0.0053) and a strong positive correlation with vascular invasion (ρ = 0.6330, p < 0.0001). No correlation was observed between PIVKA-II and the number of tumor lesions (ρ = −0.0020, p = 0.9854).

After Benjamini–Hochberg FDR correction across the 20-test family of PIVKA-II correlations with CBC parameters and ratios, RDW-CV (q = 0.031) and MLR (q = 0.031) remained statistically significant, whereas the correlation with monocyte percentage did not survive correction (q = 0.216). Within the separate three-test family of PIVKA-II correlations with tumor characteristics, tumor size (q = 0.008) and vascular invasion (q = 0.0002) remained significant after correction.

**Fig 5:**
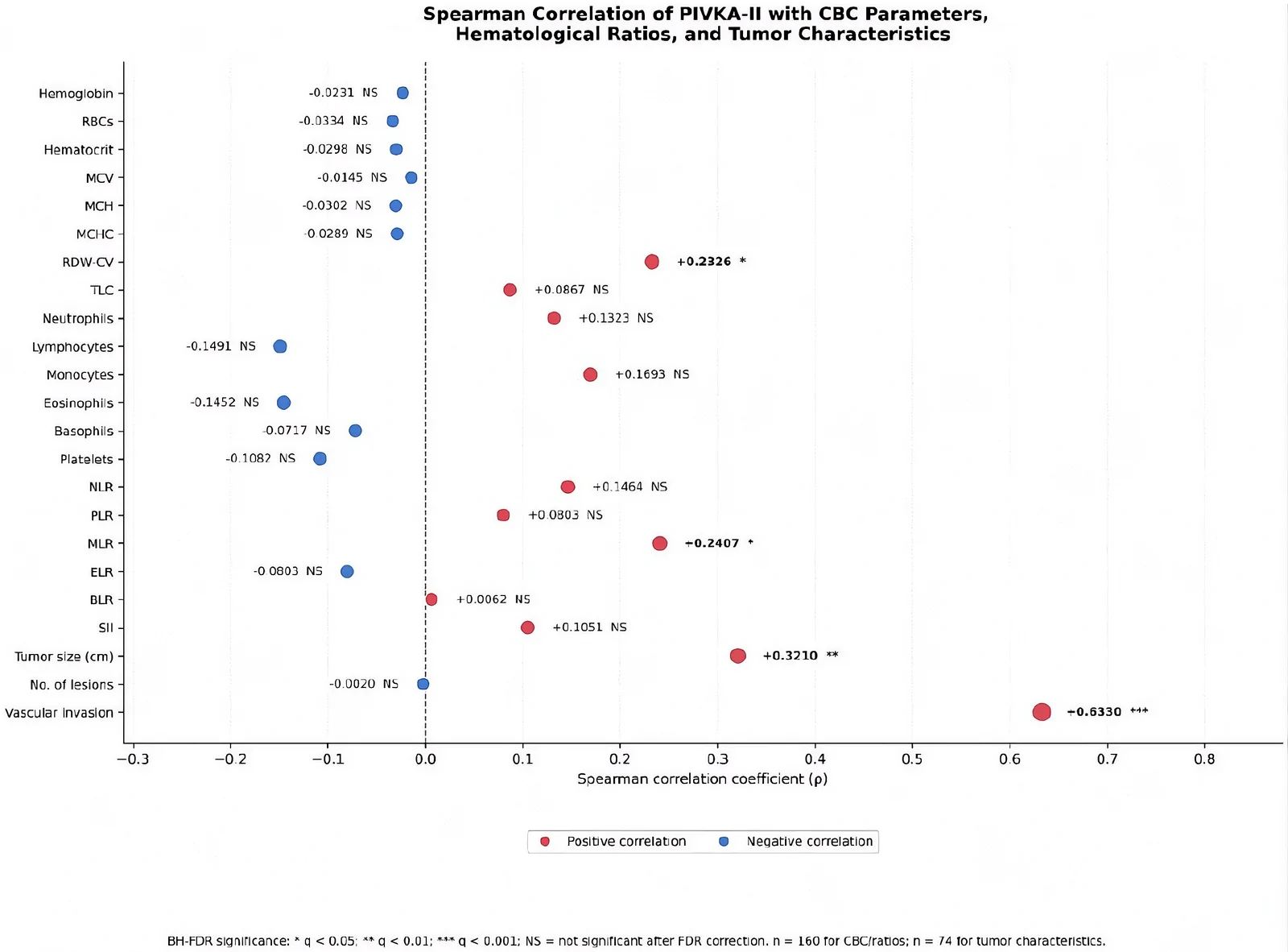
Bubble plot of Spearman correlation coefficients (ρ) between PIVKA-II and CBC/ratio parameters and Tumor characteristics. Bubble size reflects correlation magnitude. Red = positive; Blue = negative. Asterisk (*) = p < 0.05.

### Diagnostic performance of PIVKA-II, CBC parameters, and hematological ratios

ROC curve analysis was performed to evaluate the ability of PIVKA-II, CBC parameters, and hematological ratios to discriminate HCC from non-malignant CLD (Table 8; Fig 6). PIVKA-II demonstrated the highest discriminative performance, with an AUC of 0.753 (95% CI, 0.670–0.827). At the optimal cutoff of >82.1 mAU/mL, PIVKA-II yielded a sensitivity of 86.4%, specificity of 56.0%, PPV of 60.6%, NPV of 83.9%, and Youden’s J of 0.424.

**Table 8:** ROC curve analysis — diagnostic performance of PIVKA-II, CBC parameters, and hematological ratios.

*Parameter*	*AUC*	*95% CI*	*Optimal Cutoff*	*Sensitivity*	*Specificity*	*PPV*	*NPV*	*Youden’s J*
***PIVKA-II (mAU/mL)***	*0.753*	*0.670–0.827*	*>82.1 mAU/mL*	*86.4%*	*56.0%*	*60.6%*	*83.9%*	***0.424***
*Platelets (×10^9^/L)*	*0.642*	*0.561–0.730*	*<148.0*	*79.7%*	*53.5%*	*59.6%*	*75.4%*	***0.332***
*RDW/PLT Ratio*	*0.638*	*0.546–0.723*	*>0.125*	*72.6%*	*60.2%*	*61.6%*	*71.4%*	***0.328***
*Neutrophils (%)*	*0.596*	*0.509–0.683*	*>68.2%*	*56.8%*	*60.5%*	*55.3%*	*61.9%*	***0.173***
*NLR*	*0.594*	*0.505–0.680*	*>3.97*	*52.7%*	*65.1%*	*56.5%*	*61.5%*	***0.178***
*RDW-CV (%)*	*0.590*	*0.497–0.672*	*>16.0%*	*77.0%*	*40.7%*	*52.8%*	*67.3%*	***0.177***
*MLR*	*0.582*	*0.494–0.670*	*>0.505*	*55.4%*	*66.3%*	*58.6%*	*63.3%*	***0.217***
*SII*	*0.530*	*0.440–0.621*	*>265.7*	*76.7%*	*36.1%*	*51.4%*	*63.8%*	***0.128***
*PLR*	*0.510*	*0.418–0.596*	*>4.87*	*68.5%*	*41.0%*	*50.5%*	*59.6%*	***0.095***
*Lymphocytes (%)*	*0.594*	*0.505–0.682*	*<23.6%*	*74.3%*	*41.9%*	*52.3%*	*65.8%*	***0.162***

Among hematological markers, platelet count showed the second-highest discriminatory performance, with lower platelet count indicating greater likelihood of HCC (AUC = 0.642; 95% CI, 0.561–0.730). The RDW-to-platelet ratio showed a similar but slightly lower discriminatory performance (AUC = 0.638; 95% CI, 0.546–0.723). At the optimal cutoff of <148.0 × 10^9^/L, platelet count yielded a sensitivity of 79.7%, specificity of 53.5%, PPV of 59.6%, NPV of 75.4%, and Youden’s J of 0.332. At a cutoff of >0.125, the RDW-to-platelet ratio yielded a sensitivity of 72.6%, specificity of 60.2%, PPV of 61.6%, NPV of 71.4%, and Youden’s J of 0.328. Other markers demonstrated modest discriminatory ability, including neutrophil percentage (AUC = 0.596), lymphocyte percentage (AUC = 0.594), NLR (AUC = 0.594), RDW-CV (AUC = 0.590), MLR (AUC = 0.582), SII (AUC = 0.530), and PLR (AUC = 0.510).

**Fig 6:**
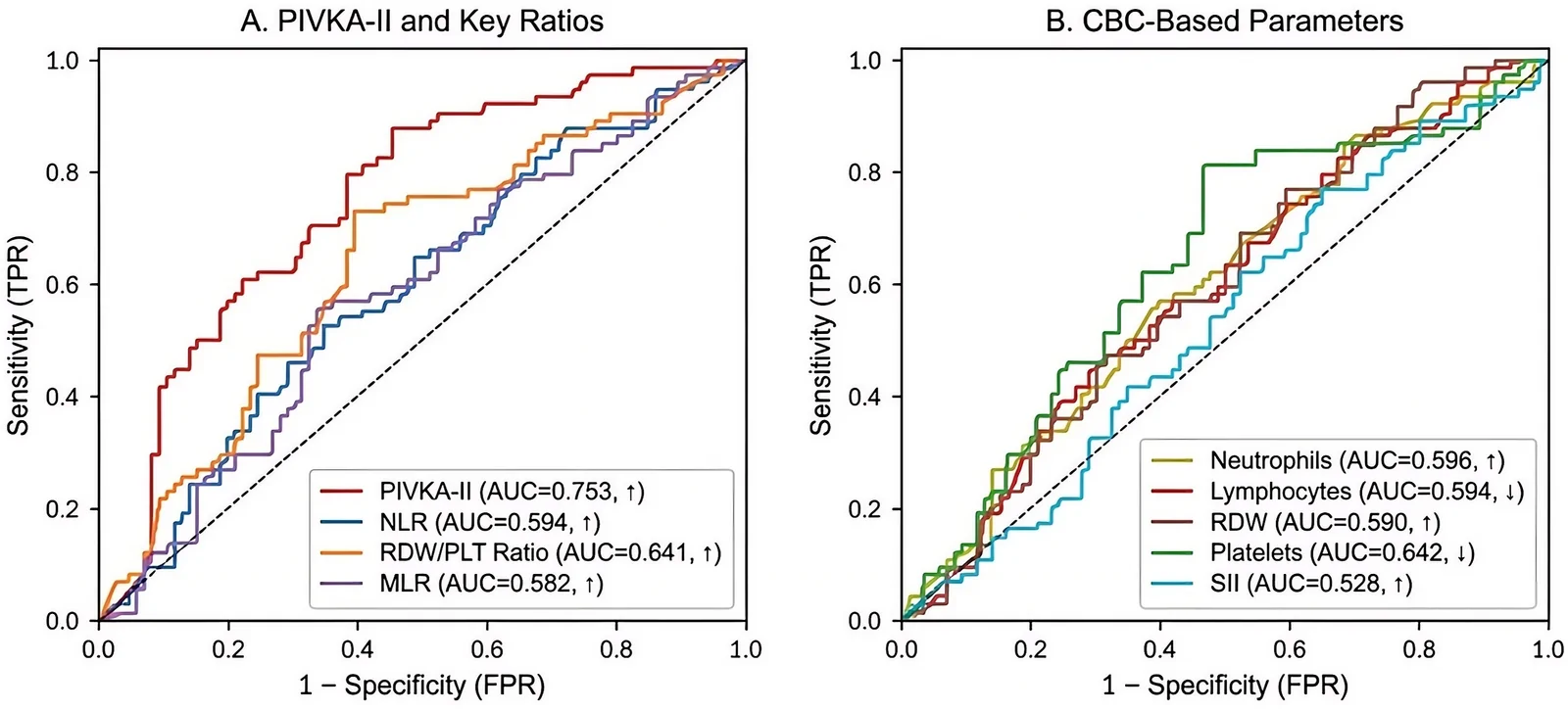
(A) ROC curves for PIVKA-II (AUC = 0.753) and key hematological ratios. (B) ROC curves for CBC-based parameters. Dashed diagonal represents no discrimination.

### Bootstrap validation and youden cut-off stability

Internal bootstrap validation using 1,000 resamples was performed to calculate optimism-corrected diagnostic metrics and evaluate cutoff stability (Table 9). PIVKA-II maintained an optimism-corrected AUC of 0.753009, compared with an apparent AUC of 0.753771, corresponding to an optimism estimate of 0.000763. The median bootstrap Youden cutoff for PIVKA-II was 141.78 mAU/mL, yielding a median sensitivity of 79.7% and specificity of 61.6%. However, the 95% percentile confidence interval for the PIVKA-II cutoff was wide (58.73–715.80 mAU/mL), indicating substantial sample-dependent variability.

**Table 9:** Bootstrap-validated diagnostic performance and youden cut-offs of PIVKA-II and hematological parameters for HCC Discrimination.

*Parameter*	*Apparent AUC*	*Bootstrap optimism*	*Optimism-corrected AUC*	*Bootstrap median Youden cutoff*	*Cutoff 95% percentile low*	*Cutoff 95% percentile high*	*Median sensitivity at bootstrap cutoff*	*Median specificity at bootstrap cutoff*
*PIVKA-II (mAU/mL)*	*0.753771*	*0.000763*	*0.753009*	*141.78*	*58.73*	*715.8*	*0.797297*	*0.616279*
*Platelets (×10^9^/L)*	*0.641578*	*−0.00023*	*0.641808*	*148*	*110*	*150*	*0.800811*	*0.534884*
*RDW/PLT Ratio*	*0.640713*	*0.000712*	*0.640001*	*0.125*	*0.116923*	*0.169474*	*0.72973*	*0.604651*
*Neutrophils (%)*	*0.595852*	*−0.00028*	*0.596136*	*66.3*	*55.6*	*81.5*	*0.594595*	*0.546512*
*NLR*	*0.593966*	*−0.00099*	*0.594958*	*3.828877*	*1.83871*	*6.745763*	*0.540541*	*0.627907*
*RDW-CV (%)*	*0.589566*	*7.55 × 10* ^−^ *^5^*	*0.589491*	*16*	*14*	*19.5*	*0.77027*	*0.406977*
*MLR*	*0.582024*	*−2.6 × 10 ⁻* ^−5^	*0.58205*	*0.504854*	*0.30303*	*0.57721*	*0.554054*	*0.662791*
*SII*	*0.528441*	*−0.00068*	*0.529126*	*269.9738*	*184.2701*	*1427.472*	*0.743243*	*0.360465*
*PLR*	*0.5077*	*−0.00017*	*0.50787*	*4.872881*	*2.067183*	*30.61224*	*0.689189*	*0.395349*
*Lymphocytes (%)*	*0.59428*	*−0.0015*	*0.595782*	*19.3*	*11.7*	*35.4*	*0.567568*	*0.581395*

Platelet count (optimism-corrected AUC = 0.641808; median cutoff = 148.0 × 10^9^/L) and the RDW-to-platelet ratio (optimism-corrected AUC = 0.640001; median cutoff = 0.125) demonstrated relatively stable bootstrap performance with percentile intervals of 110.0–150.0 × 10^9^/L and 0.117–0.169, respectively.

### 10-Fold cross-validation analysis

To evaluate whether the apparent AUCs reflected genuine discriminatory ability rather than in-sample overfitting, stratified 10-fold cross-validation (500 total train/test iterations) was performed for each marker (Table 10). For PIVKA-II, the mean cross-validated AUC was 0.751 ± 0.124 (95% range, 0.480–0.953), closely matching the apparent AUC of 0.753 (optimism = +0.002). At the training-derived cutoff of >82.1 mAU/mL, mean held-out sensitivity and specificity were 80.0% and 55.2%, respectively, consistent with the apparent estimates reported above.

**Table 10:** Fold cross-validated ROC analysis of PIVKA-II and hematological parameters for HCC discrimination.

*Parameter*	*Optimal Cutoff*	*Apparent AUC*	*10-Fold CV AUC (Mean ± SD)*	*CV AUC 95% Range*	*CV Sensitivity*	*CV Specificity*	Optimism*
***PIVKA-II (mAU/mL)***	*>82.1*	*0.753*	*0.751 ± 0.124*	*0.480–0.953*	*80.0%*	*55.2%*	*+0.002*
***Platelets (×10^9^/L)***	*<148*	*0.642*	*0.641 ± 0.137*	*0.367–0.894*	*79.6%*	*53.4%*	*+0.001*
***RDW/PLT Ratio***	*>0.125*	*0.638*	*0.640 ± 0.133*	*0.375–0.890*	*70.2%*	*60.3%*	*−0.002*
***Neutrophils (%)***	*>68.2*	*0.596*	*0.594 ± 0.137*	*0.320–0.867*	*59.9%*	*43.8%*	*+0.002*
***NLR***	*>3.97*	*0.594*	*0.594 ± 0.140*	*0.328–0.867*	*50.3%*	*53.2%*	*+0.000*
***RDW-CV (%)***	*>16.0*	*0.590*	*0.587 ± 0.134*	*0.315–0.848*	*70.0%*	*36.6%*	*+0.003*
***MLR***	*>0.505*	*0.582*	*0.581 ± 0.140*	*0.297–0.825*	*54.2%*	*64.2%*	*+0.001*
***SII***	*>265.7*	*0.530*	*0.529 ± 0.139*	*0.254–0.804*	*71.6%*	*33.1%*	*+0.001*
***PLR***	*>4.87*	*0.510*	*0.507 ± 0.138*	*0.222–0.764*	*65.9%*	*31.4%*	*+0.003*
***Lymphocytes (%)***	*<23.6*	*0.594*	*0.595 ± 0.141*	*0.328–0.873*	*53.5%*	*44.6%*	*−0.001*

Optimism was similarly minimal for platelet count (+0.001), the RDW-to-platelet ratio (−0.002), and all remaining CBC-derived parameters (range, −0.002 to +0.003), indicating none of the apparent AUCs were materially inflated by overfitting. These findings corroborate the bootstrap-based optimism correction reported above (Table 9) using an entirely independent resampling framework. As with the bootstrap analysis, the 95% cross-validated AUC ranges were wide for all markers (e.g., 0.480–0.953 for PIVKA-II), reflecting the limited precision achievable with a modest sample size when each held-out test fold contained only ~16 subjects.

### Logistic regression analysis of PIVKA-II and clinical predictors

In univariate analysis, PIVKA-II was significantly associated with HCC (OR = 1.002, 95% CI, 1.001–1.003; *p* < 0.0001). Lower platelet count (OR = 0.995, 95% CI, 0.990–1.000; *p* = 0.0316) and underlying cirrhosis (OR = 4.218, 95% CI, 1.706–10.426; *p* = 0.0018) were also significantly associated with HCC. Age, sex, hemoglobin, NLR, PLR, and RDW-to-platelet ratio were not significantly associated with HCC in univariate analysis (Table 11).

**Table 11:** Univariate logistic regression analysis of biomarkers & hematological ratios and multivariable logistic regression analysis.

*Parameter/ Variable*	*HCC Group (n = 74) Mean ± SD/ n (%)*	*Non-HCC CLD (n = 86) Mean ± SD/ n (%)*	*Unadjusted OR*	*95% CI*	*p-value*
***PIVKA-II (mAU/mL)***	*576.2 ± 710.2*	*110.1 ± 197.4*	*1.002*	*1.001–1.003*	*<0.0001*
*Age (years)*	*54.86 ± 10.56*	*55.9 ± 10.9*	*0.991*	*0.962–1.020*	*0.5435*
*Gender (Male)*	*49 (66.2%)*	*51 (59.3%)*	*1.345*	*0.705–2.566*	*0.3683*
*Hemoglobin (g/dL)*	*10.46 ± 1.77*	*9.72 ± 2.86*	*0.947*	*0.825–1.087*	*0.4377*
*Platelets (×10^9^/L)*	*120.4 ± 88.3*	*152.1 ± 87.0*	*0.995*	*0.990–1.000*	*0.0316*
*NLR*	*6.60 ± 7.22*	*5.43 ± 6.53*	*1.026*	*0.979–1.075*	*0.2875*
*PLR*	*10.85 ± 14.80*	*11.00 ± 14.27*	*0.999*	*0.978–1.021*	*0.9490*
*RDW-to-Platelet Ratio*	*0.18 ± 0.10*	*0.15 ± 0.13*	*9.881*	*0.498–196.245*	*0.1330*
***Cirrhosis, n (%)***	***67 (90.5%)***	***60 (69.8%)***	***4.218***	***1.706–10.426***	***0.0018***
***Variable/ Predictor***		***Adjusted OR (aOR)***	***95% CI***		***p-value***
***PIVKA-II (mAU/mL)***		***1.003***	***1.001–1.004***		***<0.0001***
***Platelets (×10^9^/L)***		***0.988***	***0.979–0.997***		***0.0097***
*Age (years)*		*0.981*	*0.947–1.016*		*0.2822*
*Gender (Male)*		*1.317*	*0.616–2.815*		*0.4770*
*Hemoglobin (g/dL)*		*0.960*	*0.810–1.138*		*0.6387*
*NLR*		*1.009*	*0.909–1.120*		*0.8643*
*PLR*		*1.029*	*0.978–1.084*		*0.2706*
*RDW-to-Platelet Ratio*		*0.132*	*0.001–15.020*		*0.4018*
***Cirrhosis***		***2.339***	***0.857–6.380***		***0.0970***

In the multivariable model, PIVKA-II remained independently associated with HCC (adjusted OR = 1.003, 95% CI, 1.001–1.004; *p* < 0.0001). Platelet count also remained independently associated with HCC (aOR = 0.988, 95% CI, 0.979–0.997; *p* = 0.0097). In contrast, age, sex, hemoglobin, NLR, PLR, RDW-to-platelet ratio, and cirrhosis status were not independently associated with HCC after multivariable adjustment (Table 11)

### Subgroup analysis according to tumor burden, vascular invasion, and BCLC stage

Subgroup analyses were performed to explore factors contributing to PIVKA-II diagnostic performance according to tumor burden, number of lesions, vascular invasion, and BCLC stage (Table 12, Fig 7). Diagnostic discrimination was lower among patients with tumors ≤5 cm (AUC = 0.686; 95% CI, 0.578–0.784) than among those with tumors >5 cm (AUC = 0.798; 95% CI, 0.715–0.873). PIVKA-II showed comparable discrimination in patients with a single lesion (AUC = 0.746; 95% CI, 0.525–0.929) and multiple lesions (AUC = 0.755; 95% CI, 0.674–0.830).

**Table 12:** Subgroup analysis of PIVKA-II diagnostic performance according to tumor burden and vascular invasion.

*Subgroup*	*HCC, n*	*Control, n*	*Median PIVKA-II (IQR), mAU/mL*	*PIVKA-II AUC*	*95% CI*	Sensitivity*
***Overall***	*74*	*86*	*461.6 (151–1186)*	***0.753***	*0.670–0.827*	*86.4%*
***Tumor size ≤5 cm***	*29*	*86*	*208.1 (87.6–841.5)*	***0.686***	*0.578–0.784*	*79.3%*
***Tumor size >5 cm***	*45*	*86*	*715.8 (305.1–936.0)*	***0.798***	*0.715–0.873*	*91.1%*
***Single lesion***	*8*	*86*	*818.4 (440.0–940.5)*	***0.746***	*0.525–0.929*	*75.0%*
***Multiple lesions (>*** ***1)***	*66*	*86*	*473.0 (152.7–925.9)*	***0.755***	*0.674–0.830*	*87.9%*
***Vascular invasion absent***	*31*	*86*	*149.1 (85.2–265.3)*	***0.616***	*0.511–0.717*	*77.4%*
***Vascular invasion present***	*43*	*86*	*841.5 (588.2–1000.0)*	***0.853***	*0.777–0.923*	*93.0%*
***BCLC-A***	*4*	*86*	*31.4 (26.6–47.8)*	***0.308***	*0.166–0.471*	*25.0%*
***BCLC-B***	*22*	*86*	*145.6 (96.8–239.9)*	***0.619***	*0.511–0.725*	*81.8%*
***BCLC-C***	*33*	*86*	*810.9 (473.5–950.0)*	***0.860***	*0.787–0.924*	*97.0%*
***BCLC-D***	*15*	*86*	*954.0 (753.3–1000.0)*	***0.838***	*0.693–0.948*	*86.7%*
***Early/intermediate stage (BCLC A + B)***	*26*	*86*	*142.0 (82.2–217.9)*	***0.571***	*0.463–0.670*	*73.1%*
***Advanced stage (C + D)***	*48*	*86*	*840.8 (562.2–1000.0)*	***0.853***	*0.781–0.920*	*93.8%*

The largest difference was observed according to vascular invasion. PIVKA-II showed limited discrimination among HCC patients without vascular invasion (AUC = 0.616; 95% CI, 0.511–0.717), whereas discrimination was substantially higher among those with vascular invasion (AUC = 0.853; 95% CI, 0.777–0.923). Median PIVKA-II was also markedly higher in patients with vascular invasion (841.5 mAU/mL) than in those without (149.1 mAU/mL).

Diagnostic performance varied across BCLC stages, with AUCs of 0.308 (95% CI, 0.166–0.471) for BCLC-A, 0.619 (95% CI, 0.511–0.725) for BCLC-B, 0.860 (95% CI, 0.787–0.924) for BCLC-C, and 0.838 (95% CI, 0.693–0.948) for BCLC-D. When stages were grouped, the AUC increased from 0.571 (95% CI, 0.463–0.670) in early/intermediate disease (BCLC A + B) to 0.853 (95% CI, 0.781–0.920) in advanced disease (BCLC C + D).

**Fig 7:**
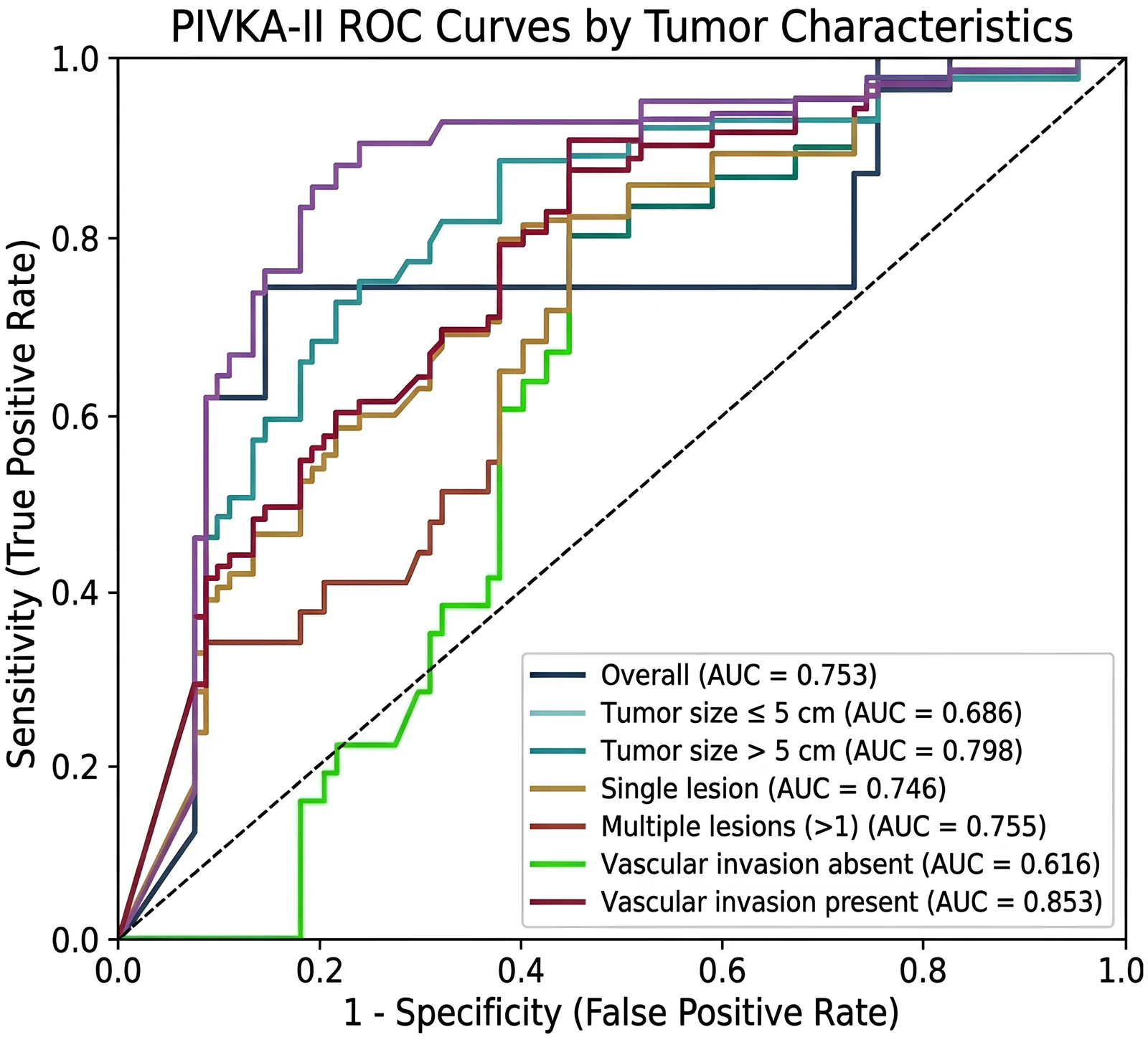
PIVKA-II ROC curves by tumor characteristics, stratified by tumor size, lesion number, and presence of vascular invasion.

### Subgroup analysis according to cirrhosis status

PIVKA-II diagnostic performance was further examined according to underlying cirrhosis status (Table 13, Fig 8). Among participants without cirrhosis, PIVKA-II yielded an AUC of 0.764 (95% CI, 0.577–0.918). The corresponding AUC was 0.615 (95% CI, 0.453–0.775) among participants with compensated cirrhosis and 0.713 (95% CI, 0.577–0.837) among those with decompensated cirrhosis.

**Table 13:** Subgroup analysis of PIVKA-II diagnostic performance according to cirrhosis status.

*Subgroup*	*HCC, n*	*Control, n*	*PIVKA-II AUC*	*95% CI*
***Overall***	*74*	*86*	***0.753***	*0.670–0.827*
***No cirrhosis***	*7*	*26*	***0.764***	*0.577–0.918*
***Compensated cirrhosis***	*15*	*33*	***0.615***	*0.453–0.775*
***Decompensated cirrhosis***	*52*	*27*	***0.713***	*0.577–0.837*
***Excluding decompensated cirrhosis from controls***	*74*	*59*	***0.830***	*0.755–0.898*

When patients with decompensated cirrhosis were excluded from the non-HCC control group, the PIVKA-II AUC increased from 0.753 to 0.830 (95% CI, 0.755–0.898), suggesting that inclusion of clinically advanced, decompensated non-malignant liver disease among controls attenuated the apparent discriminatory performance of PIVKA-II.

**Fig 8:**
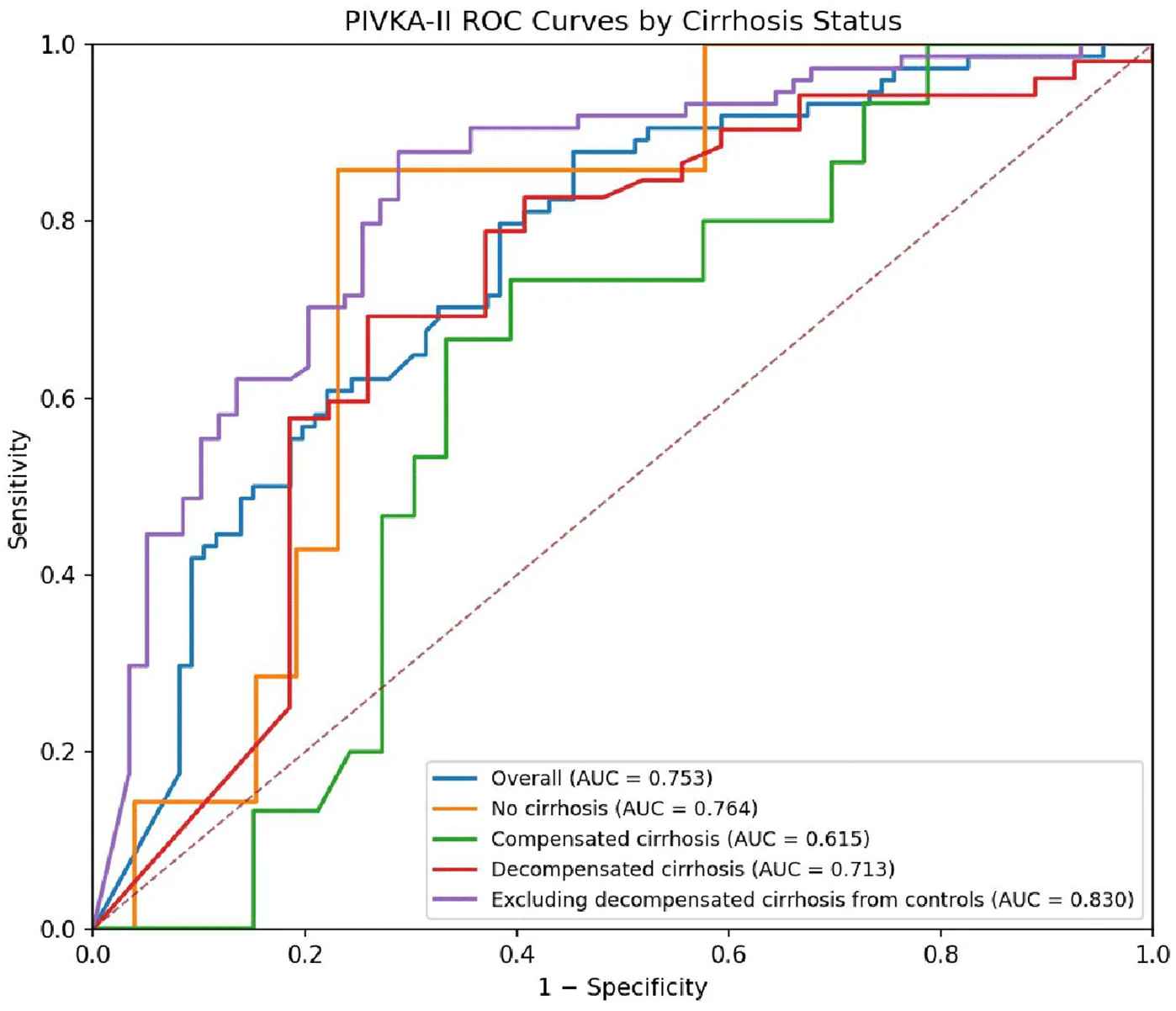
PIVKA-II ROC curves by cirrhosis status, including the analysis excluding decompensated cirrhosis from the control group.

### Stratified analysis of RDW-to-platelet ratio according to cirrhosis status

The diagnostic performance of the RDW-to-platelet ratio was assessed across cirrhosis subgroups (Table 10). Among participants without cirrhosis, the RDW-to-platelet ratio showed an AUC of 0.753, with a statistically significant difference between HCC and non-HCC CLD values (p = 0.043). The AUC was lower among participants with compensated cirrhosis (AUC = 0.567; p = 0.472) and decompensated cirrhosis (AUC = 0.583; p = 0.229). These findings indicate that the discriminatory performance of the RDW-to-platelet ratio was primarily observed in non-cirrhotic individuals and was attenuated in patients with cirrhosis (Table 14).

**Table 14:** Stratified diagnostic performance of RDW-to-platelet ratio for discriminating hepatocellular carcinoma according to cirrhosis status.

*Cirrhosis status*	*HCC, median (IQR)*	*Non-HCC CLD, median (IQR)*	*AUC (95% CI)*	*P value*
***No cirrhosis***	*0.198 (0.132–0.231)*	*0.113 (0.081–0.137)*	*0.753*	***0.043***
***Compensated cirrhosis***	*0.136 (0.111–0.165)*	*0.114 (0.095–0.165)*	*0.567*	*0.472*
***Decompensated cirrhosis***	*0.170 (0.117–0.242)*	*0.138 (0.109–0.206)*	*0.583*	*0.229*

### Comparison of diagnostic models

The diagnostic performance of PIVKA-II alone, hematological parameters alone, and a combined multivariable model is summarized in Table 15. PIVKA-II alone demonstrated an AUC of 0.753 (95% CI, 0.679–0.827), with sensitivity of 87.8%, specificity of 54.7%, PPV of 62.5%, NPV of 83.9%, and Youden’s J of 0.424.

**Table 15:** Comparison of diagnostic models for HCC: PIVKA-II Alone, hematological parameters alone, and a combined model.

*Model*	*AUC (95% CI)*	*Sensitivity*	*Specificity*	*PPV*	*NPV*	*Youden’s J*	*p vs. PIVKA-II alone*
***PIVKA-II alone***	***0.753 (0.670–0.827)***	*86.4%*	*56.0%*	*60.6%*	*83.9%*	*0.424*	*Reference*
***Hematological parameters alone (lymphocyte %, platelet count)***	***0.662 (0.582–0.755)***	*74.3%*	*54.7%*	*58.5%*	*71.2%*	*0.290*	*0.138*
***Combined (PIVKA-II + lymphocyte % + platelet count)***	***0.784 (0.720–0.859)***	*71.6%*	*73.3%*	*69.7%*	*75.0%*	*0.449*	*0.166*

The hematological model comprising lymphocyte percentage and platelet count yielded an AUC of 0.662 (95% CI, 0.582–0.755; p = 0.138 vs. PIVKA-II alone), with sensitivity of 74.3%, specificity of 54.7%, and Youden’s J of 0.290. The combined model incorporating PIVKA-II, lymphocyte percentage, and platelet count achieved the highest overall discrimination (AUC = 0.784; 95% CI, 0.720–0.859), with sensitivity of 71.6%, specificity of 73.3%, PPV of 69.7%, NPV of 75.0%, and Youden’s J of 0.449. Although the combined model demonstrated numerically greater discrimination than PIVKA-II alone, the difference did not reach statistical significance (p = 0.166).

## Discussion

This case-control study evaluated the diagnostic performance of serum PIVKA-II and routinely available hematological parameters, including the RDW-to-platelet ratio (RDW/PLT) and neutrophil-to-lymphocyte ratio (NLR), for distinguishing hepatocellular carcinoma (HCC) from non-malignant chronic liver disease (CLD) in a viral hepatitis-endemic population of Pakistan. PIVKA-II demonstrated the strongest discriminatory performance among the evaluated biomarkers, whereas platelet count and RDW/PLT showed modest discriminatory ability. Although NLR was higher in patients with HCC than in those with non-malignant CLD, its discriminatory performance was limited and the between-group difference did not remain statistically significant after correction for multiple comparisons. The combined model incorporating PIVKA-II, lymphocyte percentage, and platelet count showed a numerically higher AUC than PIVKA-II alone, but this improvement was not statistically significant. Overall, these findings support PIVKA-II as a potentially useful adjunctive biomarker for distinguishing HCC from non-malignant CLD, while suggesting that readily available hematological parameters may provide complementary clinical information rather than serving as standalone diagnostic markers.

### PIVKA-II diagnostic performance

PIVKA-II demonstrated the strongest discriminatory performance among the biomarkers evaluated in this study, with an AUC of 0.753 (95% CI, 0.670–0.827) for distinguishing HCC from non-malignant CLD. At the study-derived cut-off of >82.1 mAU/mL, sensitivity was 86.4% and specificity was 56.0%. These findings indicate fair diagnostic discrimination, characterized by relatively high sensitivity but more limited specificity in this clinically heterogeneous CLD population. The observed AUC was lower than the pooled AUC of 0.856 (95% CI, 0.817–0.895) reported by Xing et al. in a systematic review and meta-analysis of 31 studies [22]. More recent meta-analyses have also reported high pooled diagnostic performance for PIVKA-II, including an AUC of 0.89 (95% CI, 0.88–0.90) reported by Kobeissy et al. and substantial variation across clinical populations reported by Perne et al. [23, 24]. Differences in study populations, disease severity, tumor characteristics, and reference-group composition may partly explain the lower AUC observed in the present study.

The subgroup findings provide potential explanations for this difference. PIVKA-II showed lower discrimination among patients with tumors ≤5 cm than among those with tumors >5 cm (AUC, 0.686 vs. 0.798); however, this difference was relatively modest and was not statistically significant. Therefore, tumor size alone should not be considered a definitive explanation for the lower overall AUC. In contrast, PIVKA-II demonstrated substantially higher discrimination in patients with vascular invasion than in those without vascular invasion (AUC, 0.853 vs. 0.616). PIVKA-II was also positively correlated with tumor size and strongly correlated with vascular invasion after correction for multiple comparisons. These findings are consistent with previous evidence linking PIVKA-II with tumor burden and vascular invasion [25–29]. Nevertheless, the subgroup analyses were exploratory because of the relatively small number of patients in some strata and should therefore be interpreted as potential contributors to variation in diagnostic performance rather than definitive evidence of effect modification.

The BCLC-stratified analysis further suggested that PIVKA-II discrimination differed according to disease stage. The AUC was 0.571 among patients with BCLC A + B disease compared with 0.853 among those with BCLC C + D disease. This finding suggests that PIVKA-II may discriminate more effectively in advanced HCC, potentially reflecting its association with greater tumor burden and aggressive tumor characteristics [25–28]. However, the lower discrimination observed in early/intermediate disease is clinically important because the greatest value of a surveillance biomarker is its ability to identify HCC before advanced tumor characteristics develop. Therefore, the relatively high proportion of advanced-stage HCC in the present cohort may have influenced the overall diagnostic performance and limits direct extrapolation of these findings to early-stage HCC detection. It should be emphasized that strictly defined early-stage disease (BCLC-A) accounted for only 4 of 74 HCC patients (5.4%) in this cohort, and the AUC of 0.571 reported for the “early/intermediate” (BCLC A + B) subgroup therefore reflects a heterogeneous stratum in which intermediate-stage (BCLC-B, n = 22) patients predominate; this cohort could not adequately assess PIVKA-II performance in early-stage (BCLC-A) disease alone.

The cirrhosis-stratified analysis provides another potential explanation for the observed AUC. PIVKA-II yielded AUCs of 0.764 among participants without cirrhosis, 0.615 among those with compensated cirrhosis, and 0.713 among those with decompensated cirrhosis. Notably, exclusion of patients with decompensated cirrhosis from the non-HCC control group increased the AUC from 0.753 to 0.830 (95% CI, 0.755–0.898). This finding suggests that the severity and clinical composition of the CLD reference population may materially influence the apparent diagnostic performance of PIVKA-II. This interpretation is consistent with previous evidence showing that PIVKA-II performance can vary according to the underlying etiology and characteristics of chronic liver disease [29]. Thus, differences in the composition and severity of control populations should be considered when comparing PIVKA-II diagnostic performance across studies.

Internal bootstrap validation provided reassurance regarding the stability of the overall AUC. After 1,000 bootstrap resamples, the optimism-corrected AUC was 0.753, essentially unchanged from the apparent AUC of 0.753, indicating minimal estimated optimism. However, the bootstrap-derived Youden cut-off was 141.78 mAU/mL, with a wide 95% percentile range of 58.73–715.80 mAU/mL, indicating substantial sample-dependent variability in the optimal threshold. Therefore, although the overall discriminatory performance appeared internally stable, the study-derived cut-off of 82.1 mAU/mL should be considered exploratory rather than a universally applicable clinical threshold. External validation in larger independent cohorts is required before a population-specific cut-off can be recommended.

These bootstrap findings were independently corroborated by stratified 10-fold cross-validation (Table 10), a resampling approach that is methodologically distinct from bootstrapping in that it repeatedly partitions the data into non-overlapping training and test folds rather than resampling with replacement. The cross-validated AUC for PIVKA-II (0.751 ± 0.124) was nearly identical to the apparent AUC (0.753), with a similarly negligible optimism estimate (+0.002), and comparably minimal optimism was observed across all hematological parameters (range, −0.002 to +0.003). The convergence of two independent internal-validation methods strengthens confidence that the discriminatory performance reported here is not an overfitting artifact. Nonetheless, both approaches are internal validation techniques applied to the same single-center cohort; neither substitutes for external validation in an independent population before the PIVKA-II cutoff can be adopted clinically.

The combined model incorporating PIVKA-II, lymphocyte percentage, and platelet count showed a numerically higher AUC than PIVKA-II alone (0.784 vs. 0.753), with improved specificity; however, the difference was not statistically significant (p = 0.166). Accordingly, the present data do not establish a statistically significant diagnostic advantage from combining these hematological parameters with PIVKA-II. Nevertheless, the numerical improvement provides a rationale for further evaluation of multimarker approaches in larger, independently validated cohorts.

Overall, the findings support PIVKA-II as a potentially useful adjunctive biomarker for distinguishing HCC from non-malignant CLD in this hepatitis-endemic population, particularly when interpreted alongside clinical and imaging information. However, its moderate overall discrimination, lower performance in early/intermediate-stage disease, variation according to vascular invasion and cirrhosis status, and substantial variability in the optimal diagnostic threshold indicate that PIVKA-II should not be regarded as a standalone diagnostic test on the basis of the present study. The difference between the present AUC of 0.753 and the pooled estimate of 0.856 reported by Xing et al. [22] may partly reflect differences in disease stage and the severity and composition of the non-HCC reference population. Larger prospective studies with standardized reference populations and independent external validation are needed to determine the incremental clinical value of PIVKA-II and establish robust, population-specific diagnostic thresholds.

### Hematological indices and inflammatory markers

Apart from PIVKA-II, several hematological parameters differed between the HCC and non-malignant CLD groups. HCC patients had higher neutrophil percentages, lower lymphocyte percentages, lower platelet counts, and higher NLR and RDW/PLT ratios. However, after Benjamini–Hochberg false discovery rate correction, the differences in neutrophil percentage, lymphocyte percentage, and NLR were no longer statistically significant, whereas the difference in platelet count and RDW/PLT ratio remained significant. These findings therefore provide limited evidence for a specific diagnostic role of systemic inflammatory indices in distinguishing HCC from non-malignant CLD.

In particular, NLR showed only modest discriminatory performance in the present cohort (AUC = 0.594), substantially lower than that of PIVKA-II (AUC = 0.753). This finding is consistent with a prospective controlled study by Johnson et al., in which NLR demonstrated only weak diagnostic discrimination between patients with HCC and those with chronic liver disease (AUROC = 0.65, 95% CI 0.62–0.69), despite being independently associated with overall survival in HCC patients [30]. Moreover, the unadjusted difference in NLR did not remain significant after FDR correction. Accordingly, the higher NLR observed in HCC patients should be interpreted cautiously, as it may reflect the systemic inflammatory milieu associated with chronic liver disease and malignancy rather than providing strong HCC-specific diagnostic discrimination. In contrast, the prognostic relevance of NLR is supported by more extensive evidence. A recent systematic review and meta-analysis by Peng et al. demonstrated that elevated NLR was significantly associated with poorer overall survival, recurrence-free survival, and disease-free survival among patients with HCC receiving curative therapies [31].

The RDW/PLT ratio warrants a nuanced interpretation in the context of the present findings. Although RDW/PLT was significantly higher in patients with HCC than in the comparison group (median 0.154 vs. 0.116; p = 0.003) and remained statistically significant after FDR correction (q = 0.028), its overall discriminatory ability was modest (AUC = 0.638). More importantly, the cirrhosis-stratified analysis demonstrated substantial heterogeneity in diagnostic performance according to the underlying liver disease state. The AUC was relatively higher among participants without cirrhosis (0.753) but declined markedly among those with compensated cirrhosis (0.574) and decompensated cirrhosis (0.583). Thus, the apparent discriminatory capacity of RDW/PLT in the overall cohort may be partly attributable to differences in the underlying hepatic disease rather than to an HCC-specific biological signal. The substantially reduced AUC among patients with cirrhosis is particularly relevant because cirrhosis itself produces hematological changes that can alter both components of the RDW/PLT ratio.

This interpretation is consistent with previous studies demonstrating that the RDW-to-platelet ratio is associated with hepatic fibrosis and cirrhosis. Chen et al. evaluated RDW/PLT as a non-invasive index of hepatic fibrosis and cirrhosis in patients with chronic hepatitis B, while Ramzy et al. similarly demonstrated an association between RDW/PLT and fibrosis stage in patients with chronic hepatitis C [27,28]. These observations are important when interpreting the present study because they indicate that RDW/PLT is not a marker uniquely related to malignant transformation; rather, it is also substantially influenced by the severity of the underlying chronic liver disease. Consequently, an elevated RDW/PLT ratio in patients with HCC may partly reflect the coexistence or severity of fibrosis and cirrhosis, which could explain why its diagnostic discrimination was attenuated in the cirrhotic subgroups.

The results of the adjusted analysis provide further support for this interpretation. After accounting for cirrhosis status, RDW/PLT no longer demonstrated an independent association with HCC, whereas PIVKA-II retained a significant association. This differential behavior is clinically relevant because it suggests that the relationship between RDW/PLT and HCC observed in the unadjusted analysis may be largely dependent on the hepatic disease background. In other words, although RDW/PLT distinguished the HCC group from the comparison group at the unadjusted level, its association was not sufficiently independent of cirrhosis severity to support its interpretation as an HCC-specific marker. By contrast, the persistence of the PIVKA-II association after adjustment suggests that PIVKA-II provides information more directly related to the malignant process than does RDW/PLT.

The biological basis for these findings is also plausible. Platelet counts frequently decrease as chronic liver disease progresses, owing to several mechanisms, including portal hypertension with splenic sequestration, reduced hepatic thrombopoietin production, and impaired platelet production [32]. At the same time, RDW reflects heterogeneity in erythrocyte size and may be affected by chronic inflammation, nutritional deficiencies, altered erythropoiesis, and other systemic consequences of chronic liver disease. The combination of increasing RDW and decreasing platelet counts can therefore result in an elevated RDW/PLT ratio as hepatic fibrosis and cirrhosis become more advanced [32–34]. Importantly, these processes are not specific to HCC and may occur across the spectrum of chronic liver disease. This provides a plausible explanation for the reduced diagnostic performance of RDW/PLT observed in patients with compensated and decompensated cirrhosis in the present cohort.

Taken together, the present findings suggest that RDW/PLT may be more informative as an indicator of the underlying hematological and hepatic consequences of chronic liver disease than as a specific diagnostic biomarker for HCC. Its significant elevation in HCC patients should therefore not be interpreted in isolation as evidence of an HCC-specific effect. The modest overall AUC, pronounced reduction in discrimination among patients with cirrhosis, and loss of an independent association after adjustment for cirrhosis severity collectively argue against the use of RDW/PLT as a standalone diagnostic marker for HCC. Nevertheless, its association with fibrosis and cirrhosis suggests that RDW/PLT may still provide contextual information regarding the background liver disease and could potentially contribute to broader clinical assessment when interpreted alongside established HCC biomarkers. In the present cohort, however, the stronger and persistent association observed for PIVKA-II indicates that PIVKA-II has greater potential as an HCC-specific diagnostic marker, whereas the diagnostic value of RDW/PLT appears to be substantially dependent on the underlying hepatic disease state.

The correlation analysis provided additional evidence of associations between PIVKA-II and selected hematological features. PIVKA-II was positively correlated with RDW-CV (ρ = 0.2326, p = 0.0031; q = 0.031) and MLR (ρ = 0.2407, p = 0.0022; q = 0.031), whereas the correlation with absolute monocyte percentage did not remain significant after FDR correction (ρ = 0.1693, p = 0.0324; q = 0.216). LMR was also not significantly correlated with PIVKA-II. These modest associations may reflect relationships between tumor-related biological activity and systemic hematological changes; however, the relatively small correlation coefficients indicate that these relationships are weak. Because this was a cross-sectional analysis, these correlations should not be interpreted as evidence of causality or a defined mechanistic pathway.

Overall, the hematological findings suggest that NLR and RDW/PLT may reflect systemic inflammation and the hepatic consequences of chronic liver disease rather than providing strong HCC-specific diagnostic discrimination. Among these indices, RDW/PLT showed a statistically significant difference between groups, but its diagnostic performance was modest and strongly dependent on cirrhosis status. Therefore, these inexpensive parameters may have value as **supportive clinical indicators**, but the present findings do not support their use as standalone alternatives to PIVKA-II or imaging-based HCC diagnosis.

### Epidemiological context

The demographic characteristics of our study cohort — predominantly male (62.5%), with a mean age of 55.4 years, and with 90.5% of HCC patients testing anti-HCV reactive — are broadly consistent with the known epidemiological profile of HCC in Pakistan, where chronic hepatitis C has emerged as the principal driver of liver disease

[35]. Over recent decades, the country has witnessed a gradual etiological transition from HBV to HCV dominance, a shift that makes it all the more important to develop surveillance and diagnostic strategies tailored specifically to this region. The higher

anti-HCV prevalence among HCC patients compared to those with non-malignant CLD (90.5% vs. 76.7%; p = 0.020) is consistent with this epidemiological picture and underlines the critical need to scale up HCV screening and treatment efforts across Pakistan.

### Strengths of the study

This study has several strengths. First, it provides a direct comparison of PIVKA-II with routinely available hematological and inflammatory parameters, including NLR and RDW/PLT, within the same study population, allowing their diagnostic performance to be evaluated under comparable clinical conditions. Second, the study did not rely solely on comparisons of median biomarker concentrations but incorporated receiver operating characteristic analysis and AUC estimation to assess discriminatory performance. This approach demonstrated the superior diagnostic discrimination of PIVKA-II compared with the inflammatory and hematological indices examined. Third, the study incorporated stratified analyses according to clinically relevant disease characteristics, particularly cirrhosis status and BCLC stage. The substantial variation in RDW/PLT performance according to cirrhosis status and the marked improvement in PIVKA-II discrimination in advanced BCLC stages provide important insight into how underlying liver disease and tumor stage may influence biomarker performance. Fourth, the use of FDR correction reduced the likelihood that statistically significant findings among multiple biomarker comparisons were attributable to chance. Finally, multivariable analysis allowed assessment of whether the observed biomarker associations remained independent after accounting for important clinical factors, strengthening the interpretation that PIVKA-II retained a more robust association with HCC than RDW/PLT after adjustment for cirrhosis severity. Collectively, these features provide a more clinically contextualized assessment of biomarker performance rather than relying on a single unadjusted comparison.

### Limitations

This study has several limitations that should be considered when interpreting the findings. First, the case-control design and single-center recruitment from a tertiary care hospital in Sindh may limit generalizability to other populations with different etiological profiles of chronic liver disease or lower hepatitis burden. Relatedly, the positive and negative predictive values reported in Fig 6 and Table 8 were calculated at the HCC prevalence of this case-control sample (74/160, 46.3%), which is substantially higher than the prevalence expected in unselected clinical or community screening populations; because PPV and NPV are inherently prevalence-dependent, unlike sensitivity and specificity, these values are likely to differ, and probably overestimate PPV while underestimating NPV, in lower-prevalence real-world settings, and should not be extrapolated directly without re-calculation for the prevalence of the intended target population.Second the predominance of advanced-stage HCC (64.9% BCLC C/D) in the cohort likely inflated the apparent diagnostic performance of PIVKA-II relative to what might be observed in an early-detection or surveillance setting, restricting direct extrapolation of these results to early-stage disease.Strictly defined early-stage (BCLC-A) HCC comprised only 4 of 74 patients (5.4%) in this cohort, precluding a statistically robust, stage-specific evaluation of biomarker performance in early-stage disease; this small subgroup size should be considered when interpreting the applicability of these findings to early HCC surveillance. Third, although internal bootstrap validation suggested minimal optimism in the overall AUC, the wide percentile confidence interval around the optimal PIVKA-II cut-off indicates that the threshold identified here is sample-dependent and requires external validation in independent, larger cohorts before clinical adoption. This was corroborated by an independent 10-fold cross-validation analysis, which likewise showed minimal optimism (Table 10) but wide 95% cross-validated AUC ranges (e.g., 0.480–0.953 for PIVKA-II); with only ~16 subjects per held-out test fold, these cross-validated ranges should be treated as indicative rather than precise, and larger external cohorts are needed to narrow them. Finally, HCC diagnosis relied predominantly on non-invasive imaging criteria rather than universal histological confirmation, and the heterogeneous severity of liver disease among non-malignant CLD controls—particularly the inclusion of patients with decompensated cirrhosis—may have influenced the observed discriminatory performance of both PIVKA-II and the RDW-to-platelet ratio. Taken together, these limitations underscore the need for larger, multicenter, prospective studies with standardized reference populations and external validation before these biomarkers can be recommended for routine clinical use.

### Implications for clinical practice

The findings of this study have potential practical implications for the evaluation of patients with chronic liver disease in hepatitis-endemic, resource-limited settings such as Pakistan, but they should be interpreted cautiously given the study design and limited representation of early-stage HCC. PIVKA-II demonstrated moderate discriminatory ability for distinguishing HCC from non-malignant CLD, with relatively high sensitivity but only modest specificity, suggesting that it may have value as a complementary biomarker when interpreted alongside clinical assessment and appropriate imaging rather than as a stand-alone diagnostic test. Although the observed negative predictive value was 83.9% in this study, predictive values are strongly dependent on disease prevalence and therefore should not be interpreted as evidence that a low PIVKA-II concentration can reliably rule out HCC in routine clinical or population-based screening settings. Similarly, an elevated PIVKA-II concentration should be regarded as a signal warranting further clinical assessment and appropriate imaging rather than as diagnostic evidence of HCC in isolation. The stronger discrimination observed among patients with vascular invasion and advanced BCLC stage, together with the limited representation of BCLC-A disease, suggests that PIVKA-II may perform differently across the clinical spectrum of HCC and that its role in detecting early-stage disease remains uncertain. Accordingly, PIVKA-II should not replace established imaging-based diagnostic and surveillance pathways, particularly for patients with cirrhosis or those undergoing surveillance for early HCC. CBC-derived indices, including the RDW-to-platelet ratio, have the advantage of being inexpensive and routinely available; however, their overall discriminatory performance was modest and varied according to cirrhosis status, limiting their use as independent diagnostic markers. NLR likewise showed only modest discrimination and should be considered exploratory rather than a clinically established diagnostic adjunct. Importantly, the substantial variability of the bootstrap-derived PIVKA-II cut-off indicates that the threshold identified in this study should not be adopted as a fixed clinical decision point. Instead, larger multicenter prospective studies should establish and externally validate population-specific thresholds in clinically relevant surveillance and diagnostic populations, with adequate representation of early-stage HCC and standardized reference groups. Until such validation is available, PIVKA-II is best considered a complementary biomarker that may support clinical decision-making and selection for further diagnostic evaluation, but should not replace imaging, established surveillance strategies, or guideline-based diagnostic pathways for HCC.

### Comparison with existing literature

The results reported here are broadly consistent with, though not identical to, the wider body of published evidence on PIVKA-II. A Vietnamese cohort study evaluating PIVKA-II alongside AFP and the GAAD score similarly found that PIVKA-II outperformed AFP as a diagnostic marker for HCC in a hepatitis-endemic population [36], supporting the diagnostic value of PIVKA-II observed in the present study. A separate pilot study from Serbia examined PIVKA-II in patients with chronic viral hepatitis, though its focus was primarily on the **prognostic**, rather than diagnostic, utility of PIVKA-II in this population [37]; as such, it offers indirect rather than direct support for the diagnostic role emphasized here. Taken together, these studies indicate that PIVKA-II performance has now been examined across several hepatitis-endemic populations in Asia and Europe, but with meaningful heterogeneity in study design, outcome definitions, and population characteristics — reinforcing that findings should not be generalized across settings without caution.

This heterogeneity is also reflected in cut-off values. The optimal PIVKA-II threshold identified in the present study (>82.1 mAU/mL) is considerably higher than the manufacturer-recommended upper limit of normal (<40 mAU/mL), meaning that the manufacturer’s reference cutoff would flag substantially more values as abnormal than the higher, study-derived threshold, and differs from thresholds reported in other populations, consistent with the wide bootstrap-derived confidence interval (58.73–715.80 mAU/mL) observed in our internal validation. This variability — evident both across published studies and within our own bootstrap resampling — reinforces that population-specific validation is not an optional refinement but a genuine prerequisite before PIVKA-II can be responsibly incorporated into routine clinical guidelines.

## Conclusion

In conclusion, this prospective study demonstrates that PIVKA-II provides the strongest discrimination between hepatocellular carcinoma and non-malignant chronic liver disease in this hepatitis-endemic Pakistani population, with fair overall diagnostic performance (AUC 0.753) and relatively high sensitivity. However, its moderate specificity, reduced performance in early/intermediate-stage disease, variation according to cirrhosis and vascular invasion, and instability of the optimal cut-off indicate that PIVKA-II should be used as a complementary biomarker rather than a standalone diagnostic test. Platelet count and RDW/PLT were significantly associated with HCC after false discovery rate correction, but their discriminatory performance was modest, while RDW/PLT was substantially attenuated among patients with cirrhosis and did not remain independently associated with HCC after adjustment. NLR showed limited diagnostic discrimination and did not remain significant after multiple-testing correction. Although the combined PIVKA-II, lymphocyte percentage, and platelet model showed numerically improved discrimination, the improvement over PIVKA-II alone was not statistically significant. Therefore, PIVKA-II may have clinical utility as an adjunct to established imaging and clinical assessment, particularly in hepatitis-endemic and resource-limited settings, while larger multicenter studies with standardized control populations and independent external validation are needed to establish reliable population-specific thresholds and confirm its clinical utility.
