# Supplementary material for: PIVKA-II and hematological parameters in hepatocellular carcinoma and non-malignant chronic liver disease: A case-control biomarker comparison in a hepatitis-endemic population
Source: PLoS One. 2026 Nov 3;21(11):e0361802. doi: 10.1371/journal.pone.0361802 (PMC13730426; doi:10.1371/journal.pone.0361802)
Supplement: Supplementary file 1 [file pone.0361802.s002.docx]

**POINT-BY-POINT RESPONSE TO REVIEWERS**

**PIVKA-II and Hematological Parameters in Hepatocellular Carcinoma and Non-Malignant Chronic Liver Disease: A Case-Control Biomarker Comparison in a Hepatitis-Endemic Population**

**Dear Editor and Reviewers,**

We sincerely thank the Editor and Reviewers for their careful evaluation and constructive comments. We have revised the manuscript extensively to address the methodological, statistical, clinical, and reporting concerns. The study is now explicitly framed as a prospective case-control biomarker comparison rather than a population-level surveillance diagnostic-accuracy study, and the clinical interpretation has been moderated accordingly. All analyses and statements below are based on the revised manuscript.

# Reviewer #1

**Reviewer comment 1:** The authors should perform multivariable logistic regression analysis to determine whether PIVKA-II remains an independent diagnostic factor after adjusting for potential confounders.

**Response:** Thank you for this important suggestion. We performed both univariable and multivariable logistic regression analyses. In univariable analysis, PIVKA-II was significantly associated with HCC (OR=1.002, 95% CI 1.001–1.003, p<0.0001), while platelet count and cirrhosis were also associated with HCC. In the multivariable model, PIVKA-II remained independently associated with HCC (adjusted OR=1.003, 95% CI 1.001–1.004, p<0.0001), and platelet count also remained independently associated (aOR=0.988, 95% CI 0.979–0.997, p=0.0097). Age, sex, hemoglobin, NLR, PLR, RDW-to-platelet ratio, and cirrhosis were not independently associated after adjustment. These results have been added to the Results and discussed in relation to the HCC-specific versus background liver-disease effects of the biomarkers. See Table 9.

**Reviewer comment 2:** The authors should develop and compare combined diagnostic models including PIVKA-II alone, hematological parameters alone, and a combined model.

**Response:** We agree. We developed and compared three prespecified models: PIVKA-II alone; hematological parameters alone; and a combined model incorporating PIVKA-II and selected hematological parameters. For the hematological model, we specifically included **platelet count and lymphocyte percentage** because these were selected from the routine CBC parameters based on their observed between-group differences and individual discriminatory performance, while avoiding the inclusion of multiple highly correlated CBC-derived indices that could increase model complexity and overfitting. Platelet count demonstrated the strongest discriminatory performance among the CBC parameters (AUC 0.642, 95% CI 0.561–0.730), whereas lymphocyte percentage showed a modest discriminatory signal (AUC 0.594, 95% CI 0.505–0.682). Both parameters also showed lower median values in HCC patients than in non-malignant CLD controls. In contrast, several derived indices were mathematically dependent on these same CBC components, and most other CBC parameters demonstrated limited discriminatory performance.
Accordingly, the hematological-only model was restricted to these two complementary routine CBC parameters, and the same parameters were used in the combined model to provide a consistent comparison with PIVKA-II. PIVKA-II alone yielded an AUC of 0.753 (95% CI 0.670–0.827), the hematological model yielded an AUC of 0.662 (95% CI 0.582–0.755), and the combined model yielded an AUC of 0.784 (95% CI 0.720–0.859). Although the combined model showed numerically higher discrimination, the improvement over PIVKA-II alone was not statistically significant (p=0.166). We therefore do not claim a statistically proven incremental diagnostic benefit from adding the hematological parameters. The results are presented in Table 12 and are discussed as hypothesis-generating findings requiring evaluation in larger, independently validated cohorts.

**Reviewer comment 3:** More detailed clinical characteristics should be provided, including tumor size, number of lesions, BCLC stage, treatment status, cirrhosis, and comparability of age, sex, fibrosis severity, and viral hepatitis status.

**Response:** We have substantially expanded the clinical characterization. Among the 74 HCC patients, mean tumor size was 6.16±2.62 cm; 29 (39.2%) had tumors ≤5 cm and 45 (60.8%) had tumors >5 cm. The median number of lesions was 3 (IQR 2–4), with 8 (10.8%) having a single lesion and 66 (89.2%) having multiple lesions. Vascular invasion was present in 43 (58.1%). BCLC stage was A in 4 (5.4%), B in 22 (29.7%), C in 33 (44.6%), and D in 15 (20.3%); therefore, 48 (64.9%) had BCLC C/D disease. Cirrhosis was present in 67 (90.5%) HCC patients. All 74 HCC patients were newly diagnosed at enrollment and none had received HCC-directed anticancer treatment. These data are now presented in Table 2. Table 1 also reports age, sex, anti-HCV, HBsAg, and cirrhosis status for both groups. Age and sex were comparable, whereas anti-HCV reactivity and cirrhosis status differed between groups. Fibrosis stage as a separate histological variable was not available for all participants; therefore, we have not claimed direct fibrosis-stage comparability. The Discussion and Limitations now explicitly acknowledge the difference in underlying liver disease severity.

**Reviewer comment 4:** The manuscript should moderate the interpretation of NLR and RDW/platelet ratio because their AUC values are limited.

**Response:** We agree and have substantially moderated the interpretation. NLR had an AUC of 0.594 and its unadjusted between-group difference did not remain significant after Benjamini–Hochberg FDR correction (q=0.143). It is now described as exploratory and predominantly prognostic in the existing literature rather than as a reliable diagnostic marker. RDW-to-platelet ratio had an overall AUC of 0.638 and its diagnostic performance was strongly dependent on cirrhosis status; the association did not remain independently significant after multivariable adjustment. We therefore no longer describe either marker as an alternative to PIVKA-II or imaging. The revised manuscript presents them only as potentially supportive/contextual markers requiring further validation.

**Reviewer comment 5:** The optimal cut-offs were determined by the Youden index in the same dataset. The authors should perform bootstrap correction or cross-validation.

**Response:**
We thank the reviewer for this important comment. We performed **both bootstrap internal validation and repeated stratified 10-fold cross-validation** to assess the potential optimism of the ROC-derived estimates and the reproducibility of the study-derived cutoff.

First, **1,000 bootstrap resamples** were used to estimate optimism-corrected AUCs and evaluate cutoff stability. For PIVKA-II, the apparent AUC was 0.753771 and the optimism-corrected AUC was 0.753009, corresponding to minimal estimated optimism of 0.000763. However, the bootstrap median Youden cutoff was 141.78 mAU/mL, with a wide 95% percentile range of 58.73–715.80 mAU/mL, demonstrating substantial sample-dependent variability in the optimal threshold.

Second, we performed **stratified repeated 10-fold cross-validation (10 folds × 50 repeats = 500 train/test iterations)**. Importantly, in each iteration, the Youden-optimal cutoff and classification direction were determined **using only the training data (90%) and then applied without re-tuning to the corresponding held-out test fold**. For PIVKA-II, the mean held-out AUC was 0.751 ± 0.124 (95% range, 0.480–0.953), closely matching the apparent AUC of 0.753, with an optimism estimate of only +0.002. At the training-derived cutoff of >82.1 mAU/mL, the mean held-out sensitivity and specificity were 80.0% and 55.2%, respectively.

Thus, the cross-validation analysis provides an independent internal assessment showing that the **overall discriminatory performance and classification characteristics associated with the study-derived cutoff were not substantially degraded in held-out observations**. However, the wide bootstrap distribution of the optimal Youden cutoff indicates that the precise numerical threshold remains sample-dependent. Therefore, we have not interpreted the 82.1 mAU/mL value as a validated clinical cutoff. Instead, we now explicitly describe it as an **exploratory study-derived threshold** and state that an externally validated population-specific cutoff is required before clinical adoption.

The bootstrap and cross-validation analyses have been incorporated into the revised **Methods, Results, Tables 13 and 14, Discussion, Conclusion, and Limitations** sections.

**Reviewer comment 6:** Please clarify whether correction for multiple testing was applied.

**Response:** Yes. We applied the Benjamini–Hochberg procedure to prespecified families of comparisons, controlling the false discovery rate at q<0.05. Separate correction families were used for routine CBC parameters and PIVKA-II (Table 5), derived hematological ratios (Table 6), and PIVKA-II correlations (Table 7). Both nominal and FDR-adjusted p-values are reported. Findings significant only at the nominal level are explicitly interpreted as exploratory. This approach is now clearly described in the Statistical Analysis section. After FDR correction, PIVKA-II, platelet count, and RDW-to-platelet ratio remained significant; NLR did not.

# Reviewer #2

**Reviewer comment 1:** The study is effectively case-control and should be reframed explicitly as a biomarker comparison rather than a diagnostic accuracy study in a consecutive at-risk population.

**Response:** We agree. The manuscript has been explicitly reframed as a prospective case-control biomarker study. The title, Abstract, Methods, Results, Discussion, Limitations, and Conclusion now consistently describe the study as distinguishing established HCC from non-malignant CLD rather than as a population-based surveillance diagnostic-accuracy study. The Methods state that only participants classified as HCC-positive or HCC-negative before enrollment were included. We also added explicit discussion of spectrum and selection bias and clarified that diagnostic performance may differ in an unselected surveillance population containing indeterminate lesions and earlier-stage disease. The clinical implications have been moderated accordingly.

**Reviewer comment 2:** The PIVKA-II AUC is lower than pooled estimates. Provide subgroup analyses or discussion tied to tumor stage and cirrhosis burden.

**Response:** We addressed this quantitatively. PIVKA-II AUC was 0.686 for tumors ≤5 cm and 0.798 for tumors >5 cm; 0.616 without vascular invasion versus 0.853 with vascular invasion; and 0.571 for BCLC A+B versus 0.853 for BCLC C+D. Stage-specific AUCs were 0.308 for BCLC-A, 0.619 for BCLC-B, 0.860 for BCLC-C, and 0.838 for BCLC-D. PIVKA-II was positively correlated with tumor size (rho=0.321, q=0.008) and strongly with vascular invasion (rho=0.633, q=0.0002). Regarding the reference population, AUC increased from 0.753 to 0.830 (95% CI 0.755–0.898) when decompensated CLD controls were excluded. These findings suggest that tumor stage, vascular invasion, and control disease severity may contribute to differences from pooled meta-analytic estimates. We explicitly state that these subgroup analyses are exploratory and do not prove effect modification.

**Reviewer comment 3:** NLR showed only modest diagnostic performance and is primarily prognostic. Revise the discussion.

**Response:** We agree. NLR showed an AUC of 0.594 and did not remain statistically significant after FDR correction (q=0.143). The Discussion now states that NLR should be interpreted cautiously and is more strongly supported as a prognostic marker than as a diagnostic marker. We also cite the prospective controlled study by Johnson et al., in which NLR showed weak diagnostic discrimination, and a recent meta-analysis supporting its prognostic relevance. The Conclusion and Clinical Implications no longer present NLR as a reliable diagnostic adjunct.

**Reviewer comment 4:** RDW/platelet ratio has mainly been validated for fibrosis and cirrhosis. Provide stratification by cirrhosis or a clearer mechanistic discussion.

**Response:** We performed the requested cirrhosis-stratified analysis. RDW/PLT AUC was 0.753 in participants without cirrhosis, 0.567 in compensated cirrhosis, and 0.583 in decompensated cirrhosis. The ratio also showed a significant unadjusted difference overall and remained significant after FDR correction, but it was not independently associated with HCC after multivariable adjustment. The Discussion now emphasizes that RDW/PLT is strongly influenced by fibrosis/cirrhosis-related thrombocytopenia and erythrocyte-size heterogeneity and therefore should not be interpreted as an HCC-specific marker. The clinical implications have been moderated accordingly.

**Reviewer comment 5:** Optimal cut-offs were selected using the same dataset without internal validation. Perform bootstrap optimism correction or split-sample validation.

**Response:** We performed internal bootstrap validation and repeated stratified 10-fold cross-validation. Bootstrap analysis showed an optimism-corrected PIVKA-II AUC of 0.753 versus an apparent AUC of 0.753771, with minimal optimism of 0.000763. However, the bootstrap Youden cutoff showed substantial instability (median 141.78 mAU/mL; 95% percentile range 58.73–715.80). Repeated stratified 10-fold cross-validation produced a mean PIVKA-II AUC of 0.751±0.124, a 95% range of 0.480–0.953, and optimism of +0.002. We chose repeated 10-fold cross-validation rather than a single split-sample analysis because the total sample was modest and a single split would substantially reduce the effective training and testing sample sizes. We now explicitly describe both procedures and their limitations.

**Reviewer comment 6:** The manuscript should acknowledge symptom burden such as cancer pain and cite PMID 38493063.

**Response:**We added a brief statement to the Introduction acknowledging the substantial symptom burden of HCC, including cancer-related pain and its impact on quality of life and clinical management, and cited the reference corresponding to PMID 38493063. This addition provides clinical context without changing the primary biomarker research question.

**Reviewer comment 7:** Specify the manufacturer and model of the automated hematology analyzer and internal/external quality control procedures.

**Response:** We have added these details. CBC with five-part differential was performed using the Sysmex XN-550 automated hematology analyzer (Sysmex Corporation, Kobe, Japan). Internal quality control used Sysmex XN CHECK control material at appropriate concentration levels, with results monitored against manufacturer-provided targets and acceptable ranges. External analytical quality was assessed through inter-laboratory comparison of selected patient specimens analyzed independently in the study laboratory and a collaborating laboratory. These details are now included in the Laboratory Analysis section.

**Reviewer comment 8:** For PIVKA-II, report assay lot numbers and external quality control procedures, if available.

**Response:** We have added the assay lot number and quality-assurance information. PIVKA-II was measured using the Roche Cobas e 411 ECLIA system at AIMS Hyderabad; the assay lot number was 10 08273001. The analytical measurement range was 6.76–12,000 mAU/mL, with values below 40 mAU/mL considered within the manufacturer's normal range. External analytical verification was performed using reference/quality-control samples supplied through an external laboratory, with independent analysis and comparison of results. These details are now reported in the PIVKA-II Quantification section.

**Reviewer comment 9:** Define early-stage HCC more precisely using BCLC or tumor-size cutoffs and provide the number of patients.

**Response:** We have clarified the definition. Early-stage HCC is now defined strictly as BCLC-A, consistent with the BCLC staging framework. In this cohort, only 4 of 74 HCC patients (5.4%) were BCLC-A. BCLC-B was classified as intermediate stage. The previously reported BCLC A+B subgroup (n=26) is explicitly identified as early/intermediate rather than early-stage disease and is not equated with BCLC-A. Because only four BCLC-A cases were available, a statistically reliable stage-specific estimate for early HCC could not be derived. This clarification appears in the Methods/Results, Discussion, Limitations, and clinical implications.

**Reviewer comment 10:** The data availability statements are inconsistent and should be harmonized.

**Response:** We harmonized the Data Availability Statement throughout the manuscript and submission materials to the following wording: “All relevant data underlying the findings of this study are not deposited in a public repository because of patient privacy considerations and institutional data governance requirements at the University of Sindh, Jamshoro. Anonymised datasets supporting the reported analyses are available from the corresponding author upon reasonable request, subject to a data-sharing agreement with the University of Sindh, Jamshoro.”

**Reviewer comment 11:** Use a single consistent expansion of PIVKA-II: Protein Induced by Vitamin K Absence or Antagonist-II.

**Response:** We have standardized the full expansion of PIVKA-II throughout the manuscript to “Protein Induced by Vitamin K Absence or Antagonist-II” and retained the abbreviation PIVKA-II thereafter.

**Reviewer comment 12:** Use ×10⁹/L consistently instead of interchangeably using ×10³/μL and ×10⁹/L.

**Response:** We have standardized the platelet and leukocyte concentration units throughout the manuscript and tables to ×10⁹/L, including the CBC tables, ROC analyses, regression tables, and description of derived indices.

**Reviewer comment 13:** PPV and NPV are derived from the case-control prevalence and may not be generalizable. Explicitly acknowledge this limitation.

**Response:** We agree and have added an explicit limitation. The study contained 74 HCC cases among 160 participants (46.3%), which is substantially higher than the HCC prevalence expected in many unselected clinical or community screening populations. We now state that PPV and NPV are prevalence-dependent and should not be extrapolated directly to lower-prevalence populations; PPV would generally decrease and NPV increase as prevalence decreases, assuming sensitivity and specificity remain similar. The Clinical Implications section also explicitly states that the observed NPV of 83.9% should not be interpreted as evidence that a low PIVKA-II value can reliably rule out HCC in routine surveillance.

**Author-identified correction during revision:**
During the revision process, we conducted an additional verification of the ROC analyses for all continuous hematological parameters. This quality-control review identified an inconsistency in the classification direction for platelet count and lymphocyte percentage in the original analysis. Although this issue was **not raised by the reviewer**, we considered it important to correct it to ensure that the ROC classification direction was consistent with the observed association with HCC.

In the revised analysis, lower platelet counts and lower lymphocyte percentages are correctly represented as the direction associated with HCC discrimination. Platelet count showed an AUC of 0.642 (95% CI: 0.561–0.730), with an optimal cutoff of <148.0 × 10⁹/L, sensitivity of 79.7%, and specificity of 53.5%. Lymphocyte percentage showed an AUC of 0.594 (95% CI: 0.505–0.682), with an optimal cutoff of <23.6%, sensitivity of 74.3%, and specificity of 41.9%.

The correction affects the ROC classification direction and corresponding cutoff interpretation but does not change the underlying AUC estimates. The corrected direction and cutoffs have been updated consistently in the relevant tables, Results, and interpretation. We also re-checked the cross-validation procedure to ensure that the classification direction and Youden-optimal cutoff were determined from the training data only and subsequently applied unchanged to the corresponding held-out test data.

We have made this correction proactively during the revision process to improve the statistical accuracy and internal consistency of the manuscript.
