# Supplementary material for: PIVKA-II and hematological parameters in hepatocellular carcinoma and non-malignant chronic liver disease: A case-control biomarker comparison in a hepatitis-endemic population
Source: PLoS One. 2026 Nov 3;21(11):e0361802. doi: 10.1371/journal.pone.0361802 (PMC13730426; doi:10.1371/journal.pone.0361802)
Supplement: Peer review history

# Peer review: Decision Letter 0

**DOI:** 10.1371/journal.pone.0361802.r001

**Authors:** Chen Ling

14 Aug 2026

PONE-D-26-21745

Diagnostic Performance of Protein Induced by Vitamin K Absence-II, Hematological Ratios, and Complete Blood Count Parameters in Distinguishing Hepatocellular Carcinoma from Non-Malignant Chronic Liver Disease in a Hepatitis-Endemic Population

PLOS One

Dear Dr. Hashmi,

Thank you for submitting your manuscript to PLOS ONE. After careful consideration, we feel that it has merit but does not fully meet PLOS ONE’s publication criteria as it currently stands. Therefore, we invite you to submit a revised version of the manuscript that addresses the points raised during the review process.

Please submit your revised manuscript by Oct 13 2026 11:59PM. If you will need more time than this to complete your revisions, please reply to this message or contact the journal office at plosone@plos.org. When you're ready to submit your revision, log on to https://www.editorialmanager.com/pone/ and select the 'Submissions Needing Revision' folder to locate your manuscript file.

Please include the following items when submitting your revised manuscript:

- A letter that responds to each point raised by the academic editor and reviewer(s). You should upload this letter as a separate file labeled 'Response to Reviewers'.
- A marked-up copy of your manuscript that highlights changes made to the original version. You should upload this as a separate file labeled 'Revised Manuscript with Track Changes'.
- An unmarked version of your revised paper without tracked changes. You should upload this as a separate file labeled 'Manuscript'.

If you would like to make changes to your financial disclosure, please include your updated statement in your cover letter. Guidelines for resubmitting your figure files are available below the reviewer comments at the end of this letter.

If applicable, we recommend that you deposit your laboratory protocols in protocols.io to enhance the reproducibility of your results. Protocols.io assigns your protocol its own identifier (DOI) so that it can be cited independently in the future. For instructions see: https://journals.plos.org/plosone/s/submission-guidelines#loc-laboratory-protocols. Additionally, PLOS ONE offers an option for publishing peer-reviewed Lab Protocol articles, which describe protocols hosted on protocols.io. Read more information on sharing protocols at https://plos.org/protocols?utm_medium=editorial-email&utm_source=authorletters&utm_campaign=protocols.

As the corresponding author, your ORCID iD is verified in the submission system and will appear in the published article. PLOS supports the use of ORCID, and we encourage all coauthors to register for an ORCID iD and use it as well. Please encourage your coauthors to verify their ORCID iD within the submission system before final acceptance, as unverified ORCID iDs will not appear in the published article. Only the individual author can complete the verification step; PLOS staff cannot verify ORCID iDs on behalf of authors.

We look forward to receiving your revised manuscript.

Kind regards,

Chen Ling, Ph.D.

Academic Editor

PLOS One

Journal Requirements:

When submitting your revision, we need you to address these additional requirements.

1. Please ensure that your manuscript meets PLOS ONE's style requirements, including those for file naming. The PLOS ONE style templates can be found at

https://journals.plos.org/plosone/s/file?id=wjVg/PLOSOne_formatting_sample_main_body.pdf and

https://journals.plos.org/plosone/s/file?id=ba62/PLOSOne_formatting_sample_title_authors_affiliations.pdf

2. Please include your full ethics statement in the ‘Methods’ section of your manuscript file. In your statement, please include the full name of the IRB or ethics committee who approved or waived your study, as well as whether or not you obtained informed written or verbal consent. If consent was waived for your study, please include this information in your statement as well

3. In the online submission form, you indicated that "The data underlying the results presented in this study are available from the corresponding author upon reasonable request. Due to ethical and institutional restrictions related to patient confidentiality, the data are not publicly available. De-identified data can be shared with qualified researchers following approval from the Institutional Ethics Committee of the University of Sindh, Jamshoro."

All PLOS journals now require all data underlying the findings described in their manuscript to be freely available to other researchers, either 1. In a public repository, 2. Within the manuscript itself, or 3. Uploaded as supplementary information.

This policy applies to all data except where public deposition would breach compliance with the protocol approved by your research ethics board. If your data cannot be made publicly available for ethical or legal reasons (e.g., public availability would compromise patient privacy), please explain your reasons on resubmission and your exemption request will be escalated for approval.

4. If the reviewer comments include a recommendation to cite specific previously published works, please review and evaluate these publications to determine whether they are relevant and should be cited. There is no requirement to cite these works unless the editor has indicated otherwise.

[Note: HTML markup is below. Please do not edit.]

Reviewers' comments:

Reviewer's Responses to Questions

**Comments to the Author**

1. Is the manuscript technically sound, and do the data support the conclusions?

Reviewer #1: Partly

Reviewer #2: Yes

**********

2. Has the statistical analysis been performed appropriately and rigorously?

Reviewer #1: Yes

Reviewer #2: Yes

**********

3. Have the authors made all data underlying the findings in their manuscript fully available?

The PLOS Data policy

Reviewer #1: Yes

Reviewer #2: Yes

**********

4. Is the manuscript presented in an intelligible fashion and written in standard English?

Reviewer #1: Yes

Reviewer #2: Yes

**********

Reviewer #1: The manuscript addresses an important clinical question regarding the diagnostic value of PIVKA-II and RDW/platelet ratio for distinguishing HCC from non-malignant chronic liver disease. The topic is relevant; however, several issues need to be addressed before further consideration.

1. The current study mainly compares individual biomarkers between HCC and non-malignant CLD groups. However, the development of HCC is influenced by multiple clinical factors. The authors should perform multivariable logistic regression analysis to determine whether PIVKA-II remains an independent diagnostic factor after adjusting for potential confounders.

2. The authors should consider developing and comparing combined diagnostic models, including: (1) PIVKA-II alone, (2) hematological parameters alone, and (3) a combined model incorporating both PIVKA-II and hematological indicators. Comparison of their diagnostic performance would provide stronger evidence regarding the value of hematological markers.

3. More detailed clinical characteristics of the HCC patients should be provided, including tumor size, number of lesions, and BCLC stage. The authors should also clarify whether HCC patients were newly diagnosed cases or previously diagnosed patients, whether patients were receiving anticancer treatments, and the proportion of HCC patients with cirrhosis. Whether the two groups were comparable regarding important factors such as age, sex, fibrosis severity, and viral hepatitis status? These data are essential to determine whether the diagnostic performance of PIVKA-II reflects early HCC detection or mainly reflects discrimination of advanced disease.

4. The manuscript suggests that NLR and RDW/platelet ratio could serve as affordable alternatives when PIVKA-II testing is unavailable. However, the reported AUC values of these markers are relatively limited and may not fully support this conclusion. The authors should moderate this interpretation.

5. The optimal cut-off values were determined using the Youden index within the same dataset that was used for performance evaluation. This approach may lead to overestimation of diagnostic accuracy. The authors should perform internal validation approaches, such as bootstrap correction or cross-validation, to provide more reliable estimates of diagnostic performance.

6. Multiple comparisons were performed among numerous CBC parameters and hematological indices. The authors should clarify whether any correction for multiple testing (e.g., Bonferroni correction or false discovery rate adjustment) was applied.

Reviewer #2: This single-center, prospective cross-sectional study from Pakistan enrolled 160 adults with chronic liver disease 74 HCC, 86 non-malignant CLD to evaluate serum PIVKA-II and CBC-derived inflammatory indices (NLR, PLR, MLR, SII, RDW/platelet ratio) for distinguishing HCC from non-malignant CLD. PIVKA-II was significantly higher in HCC (median 461.6 vs 59.6 mAU/mL; p<0.001) and achieved an AUC of 0.753 (cut-off >82.1 mAU/mL: sensitivity 86.4%, specificity 56.0%). NLR and RDW/platelet ratio were also elevated in HCC but showed only modest discrimination (AUC 0.594 and 0.638, respectively). The authors conclude that PIVKA-II has fair diagnostic accuracy in this hepatitis-endemic setting and that CBC ratios may serve as low-cost adjuncts.

Major Comments

1. The study design is effectively case-control, as all patients were enrolled only after being classified as HCC-positive or HCC-negative by imaging or histology. This makes it a biomarker comparison in known cases rather than a diagnostic accuracy study in a consecutive at-risk population. The authors should reframe the study explicitly as a case-control biomarker comparison and temper the clinical claims accordingly.

2. The observed AUC for PIVKA-II in this study is 0.753, which is lower than the pooled estimate of approximately 0.856 reported in prior meta-analyses. The manuscript mentions this discrepancy but does not explore it quantitatively. The authors should provide subgroup analyses or a more detailed discussion tied to Figure 6 to explain potential sources of this difference, such as tumor stage distribution or cirrhosis burden in controls.

3. NLR showed only modest diagnostic performance with an AUC of 0.594. Existing evidence indicates that NLR is primarily a prognostic rather than a diagnostic marker. The authors should revise the discussion to align their diagnostic claims for NLR with the published literature.

4. The RDW/platelet ratio has been mainly validated as a non-invasive marker of hepatic fibrosis in prior studies. Although it was elevated in the HCC group, the authors have not demonstrated that its diagnostic value is independent of fibrosis or cirrhosis severity. They should provide analyses stratified by fibrosis stage or cirrhosis status, or at least offer a clearer mechanistic discussion linked to Figure 3.

5. Optimal cut-offs were selected by maximizing Youden's index on the same dataset used for performance estimation, with no internal validation. The authors should perform bootstrap optimism correction or split-sample validation and report bias-corrected AUCs and cut-offs.

6. The authors acknowledge in the discussion that tumor size, nodule number, vascular invasion, and BCLC stage were not reported. Since PIVKA-II levels correlate with tumor burden, the authors should provide subgroup analyses or correlations between PIVKA-II and imaging-based tumor burden metrics.

Minor Comments

1. In the Introduction, the authors have effectively established the high mortality and burden of HCC, particularly in regions endemic with viral hepatitis. To further strengthen the contextualization of HCC as a multifaceted disease, it would be valuable to briefly acknowledge the significant symptom burden (e.g., cancer pain) that patients experience, which often complicates their clinical management and diminishes their quality of life. PMID: 38493063 could be cited.

2. In the Methods section, please specify the manufacturer and model of the automated hematology analyzer used for CBC and briefly describe internal and external quality control procedures.

3. For PIVKA-II quantification, please report the assay lot numbers and describe any external quality control procedures, if available.

4. Please define "early-stage HCC" more precisely using BCLC stage or tumor size cutoffs, and provide the number of patients in this category.

5. The data availability statements on page 5 of the submission form, page 8 of the title page, and page 28 of the manuscript are inconsistent. Please harmonize them to ensure that conditions for access and repository status are stated uniformly.

6. The full expansion of PIVKA-II differs between page 1 and page 10. Please use a single consistent expansion throughout: "Protein Induced by Vitamin K Absence or Antagonist-II".

7. The units "×10³/μL" and "×10⁹/L" are used interchangeably in the text and tables. Please adopt "×10⁹/L" consistently throughout.

8. The PPV and NPV reported in Figure 6 and Table 7 are derived from the prevalence in this case-control sample and may not be generalizable. The authors should explicitly acknowledge this limitation in the discussion.

**********

what does this mean?). If published, this will include your full peer review and any attached files.

If you choose “no”, your identity will remain anonymous but your review may still be made public.

**Do you want your identity to be public for this peer review?**  For information about this choice, including consent withdrawal, please see our Privacy Policy

Reviewer #1: No

Reviewer #2: No

**********

[NOTE: If reviewer comments were submitted as an attachment file, they will be attached to this email and accessible via the submission site. Please log into your account, locate the manuscript record, and check for the action link "View Attachments". If this link does not appear, there are no attachment files.]

To ensure your figures meet our technical requirements, please review our figure guidelines: https://journals.plos.org/plosone/s/figures

You may also use PLOS’s free figure tool, NAAS, to help you prepare publication quality figures: https://journals.plos.org/plosone/s/figures#loc-tools-for-figure-preparation.

NAAS will assess whether your figures meet our technical requirements by comparing each figure against our figure specifications.

# Author comment: Author response to Decision Letter 1

**DOI:** 10.1371/journal.pone.0361802.r002

26 Sep 2026

POINT-BY-POINT RESPONSE TO REVIEWERS

PIVKA-II and Hematological Parameters in Hepatocellular Carcinoma and Non-Malignant Chronic Liver Disease: A Case-Control Biomarker Comparison in a Hepatitis-Endemic Population

Dear Editor and Reviewers,

We sincerely thank the Editor and Reviewers for their careful evaluation and constructive comments. We have revised the manuscript extensively to address the methodological, statistical, clinical, and reporting concerns. The study is now explicitly framed as a prospective case-control biomarker comparison rather than a population-level surveillance diagnostic-accuracy study, and the clinical interpretation has been moderated accordingly. All analyses and statements below are based on the revised manuscript.

Reviewer #1

Reviewer comment 1: The authors should perform multivariable logistic regression analysis to determine whether PIVKA-II remains an independent diagnostic factor after adjusting for potential confounders.

Response: Thank you for this important suggestion. We performed both univariable and multivariable logistic regression analyses. In univariable analysis, PIVKA-II was significantly associated with HCC (OR=1.002, 95% CI 1.001–1.003, p<0.0001), while platelet count and cirrhosis were also associated with HCC. In the multivariable model, PIVKA-II remained independently associated with HCC (adjusted OR=1.003, 95% CI 1.001–1.004, p<0.0001), and platelet count also remained independently associated (aOR=0.988, 95% CI 0.979–0.997, p=0.0097). Age, sex, hemoglobin, NLR, PLR, RDW-to-platelet ratio, and cirrhosis were not independently associated after adjustment. These results have been added to the Results and discussed in relation to the HCC-specific versus background liver-disease effects of the biomarkers. See Table 9.

Reviewer comment 2: The authors should develop and compare combined diagnostic models including PIVKA-II alone, hematological parameters alone, and a combined model.

Response: We agree. We developed and compared three prespecified models: PIVKA-II alone; hematological parameters alone; and a combined model incorporating PIVKA-II and selected hematological parameters. For the hematological model, we specifically included platelet count and lymphocyte percentage because these were selected from the routine CBC parameters based on their observed between-group differences and individual discriminatory performance, while avoiding the inclusion of multiple highly correlated CBC-derived indices that could increase model complexity and overfitting. Platelet count demonstrated the strongest discriminatory performance among the CBC parameters (AUC 0.642, 95% CI 0.561–0.730), whereas lymphocyte percentage showed a modest discriminatory signal (AUC 0.594, 95% CI 0.505–0.682). Both parameters also showed lower median values in HCC patients than in non-malignant CLD controls. In contrast, several derived indices were mathematically dependent on these same CBC components, and most other CBC parameters demonstrated limited discriminatory performance.

Accordingly, the hematological-only model was restricted to these two complementary routine CBC parameters, and the same parameters were used in the combined model to provide a consistent comparison with PIVKA-II. PIVKA-II alone yielded an AUC of 0.753 (95% CI 0.670–0.827), the hematological model yielded an AUC of 0.662 (95% CI 0.582–0.755), and the combined model yielded an AUC of 0.784 (95% CI 0.720–0.859). Although the combined model showed numerically higher discrimination, the improvement over PIVKA-II alone was not statistically significant (p=0.166). We therefore do not claim a statistically proven incremental diagnostic benefit from adding the hematological parameters. The results are presented in Table 12 and are discussed as hypothesis-generating findings requiring evaluation in larger, independently validated cohorts.

Reviewer comment 3: More detailed clinical characteristics should be provided, including tumor size, number of lesions, BCLC stage, treatment status, cirrhosis, and comparability of age, sex, fibrosis severity, and viral hepatitis status.

Response: We have substantially expanded the clinical characterization. Among the 74 HCC patients, mean tumor size was 6.16±2.62 cm; 29 (39.2%) had tumors ≤5 cm and 45 (60.8%) had tumors >5 cm. The median number of lesions was 3 (IQR 2–4), with 8 (10.8%) having a single lesion and 66 (89.2%) having multiple lesions. Vascular invasion was present in 43 (58.1%). BCLC stage was A in 4 (5.4%), B in 22 (29.7%), C in 33 (44.6%), and D in 15 (20.3%); therefore, 48 (64.9%) had BCLC C/D disease. Cirrhosis was present in 67 (90.5%) HCC patients. All 74 HCC patients were newly diagnosed at enrollment and none had received HCC-directed anticancer treatment. These data are now presented in Table 2. Table 1 also reports age, sex, anti-HCV, HBsAg, and cirrhosis status for both groups. Age and sex were comparable, whereas anti-HCV reactivity and cirrhosis status differed between groups. Fibrosis stage as a separate histological variable was not available for all participants; therefore, we have not claimed direct fibrosis-stage comparability. The Discussion and Limitations now explicitly acknowledge the difference in underlying liver disease severity.

Reviewer comment 4: The manuscript should moderate the interpretation of NLR and RDW/platelet ratio because their AUC values are limited.

Response: We agree and have substantially moderated the interpretation. NLR had an AUC of 0.594 and its unadjusted between-group difference did not remain significant after Benjamini–Hochberg FDR correction (q=0.143). It is now described as exploratory and predominantly prognostic in the existing literature rather than as a reliable diagnostic marker. RDW-to-platelet ratio had an overall AUC of 0.638 and its diagnostic performance was strongly dependent on cirrhosis status; the association did not remain independently significant after multivariable adjustment. We therefore no longer describe either marker as an alternative to PIVKA-II or imaging. The revised manuscript presents them only as potentially supportive/contextual markers requiring further validation.

Reviewer comment 5: The optimal cut-offs were determined by the Youden index in the same dataset. The authors should perform bootstrap correction or cross-validation.

Response:

We thank the reviewer for this important comment. We performed both bootstrap internal validation and repeated stratified 10-fold cross-validation to assess the potential optimism of the ROC-derived estimates and the reproducibility of the study-derived cutoff.

First, 1,000 bootstrap resamples were used to estimate optimism-corrected AUCs and evaluate cutoff stability. For PIVKA-II, the apparent AUC was 0.753771 and the optimism-corrected AUC was 0.753009, corresponding to minimal estimated optimism of 0.000763. However, the bootstrap median Youden cutoff was 141.78 mAU/mL, with a wide 95% percentile range of 58.73–715.80 mAU/mL, demonstrating substantial sample-dependent variability in the optimal threshold.

Second, we performed stratified repeated 10-fold cross-validation (10 folds × 50 repeats = 500 train/test iterations). Importantly, in each iteration, the Youden-optimal cutoff and classification direction were determined using only the training data (90%) and then applied without re-tuning to the corresponding held-out test fold. For PIVKA-II, the mean held-out AUC was 0.751 ± 0.124 (95% range, 0.480–0.953), closely matching the apparent AUC of 0.753, with an optimism estimate of only +0.002. At the training-derived cutoff of >82.1 mAU/mL, the mean held-out sensitivity and specificity were 80.0% and 55.2%, respectively.

Thus, the cross-validation analysis provides an independent internal assessment showing that the overall discriminatory performance and classification characteristics associated with the study-derived cutoff were not substantially degraded in held-out observations. However, the wide bootstrap distribution of the optimal Youden cutoff indicates that the precise numerical threshold remains sample-dependent. Therefore, we have not interpreted the 82.1 mAU/mL value as a validated clinical cutoff. Instead, we now explicitly describe it as an exploratory study-derived threshold and state that an externally validated population-specific cutoff is required before clinical adoption.

The bootstrap and cross-validation analyses have been incorporated into the revised Methods, Results, Tables 13 and 14, Discussion, Conclusion, and Limitations sections.

Reviewer comment 6: Please clarify whether correction for multiple testing was applied.

Response: Yes. We applied the Benjamini–Hochberg procedure to prespecified families of comparisons, controlling the false discovery rate at q<0.05. Separate correction families were used for routine CBC parameters and PIVKA-II (Table 5), derived hematological ratios (Table 6), and PIVKA-II correlations (Table 7). Both nominal and FDR-adjusted p-values are reported. Findings significant only at the nominal level are explicitly interpreted as exploratory. This approach is now clearly described in the Statistical Analysis section. After FDR correction, PIVKA-II, platelet count, and RDW-to-platelet ratio remained significant; NLR did not.

Reviewer #2

Reviewer comment 1: The study is effectively case-control and should be reframed explicitly as a biomarker comparison rather than a diagnostic accuracy study in a consecutive at-risk population.

Response: We agree. The manuscript has been explicitly reframed as a prospective case-control biomarker study. The title, Abstract, Methods, Results, Discussion, Limitations, and Conclusion now consistently describe the study as distinguishing established HCC from non-malignant CLD rather than as a population-based surveillance diagnostic-accuracy study. The Methods state that only participants classified as HCC-positive or HCC-negative before enrollment were included. We also added explicit discussion of spectrum and selection bias and clarified that diagnostic performance may differ in an unselected surveillance population containing indeterminate lesions and earlier-stage disease. The clinical implications have been moderated accordingly.

Reviewer comment 2: The PIVKA-II AUC is lower than pooled estimates. Provide subgroup analyses or discussion tied to tumor stage and cirrhosis burden.

Response: We addressed this quantitatively. PIVKA-II AUC was 0.686 for tumors ≤5 cm and 0.798 for tumors >5 cm; 0.616 without vascular invasion versus 0.853 with vascular invasion; and 0.571 for BCLC A+B versus 0.853 for BCLC C+D. Stage-specific AUCs were 0.308 for BCLC-A, 0.619 for BCLC-B, 0.860 for BCLC-C, and 0.838 for BCLC-D. PIVKA-II was positively correlated with tumor size (rho=0.321, q=0.008) and strongly with vascular invasion (rho=0.633, q=0.0002). Regarding the reference population, AUC increased from 0.753 to 0.830 (95% CI 0.755–0.898) when decompensated CLD controls were excluded. These findings suggest that tumor stage, vascular invasion, and control disease severity may contribute to differences from pooled meta-analytic estimates. We explicitly state that these subgroup analyses are exploratory and do not prove effect modification.

Reviewer comment 3: NLR showed only modest diagnostic performance and is primarily prognostic. Revise the discussion.

Response: We agree. NLR showed an AUC of 0.594 and did not remain statistically significant after FDR correction (q=0.143). The Discussion now states that NLR should be interpreted cautiously and is more strongly supported as a prognostic marker than as a diagnostic marker. We also cite the prospective controlled study by Johnson et al., in which NLR showed weak diagnostic discrimination, and a recent meta-analysis supporting its prognostic relevance. The Conclusion and Clinical Implications no longer present NLR as a reliable diagnostic adjunct.

Reviewer comment 4: RDW/platelet ratio has mainly been validated for fibrosis and cirrhosis. Provide stratification by cirrhosis or a clearer mechanistic discussion.

Response: We performed the requested cirrhosis-stratified analysis. RDW/PLT AUC was 0.753 in participants without cirrhosis, 0.567 in compensated cirrhosis, and 0.583 in decompensated cirrhosis. The ratio also showed a significant unadjusted difference overall and remained significant after FDR correction, but it was not independently associated with HCC after multivariable adjustment. The Discussion now emphasizes that RDW/PLT is strongly influenced by fibrosis/cirrhosis-related thrombocytopenia and erythrocyte-size heterogeneity and therefore should not be interpreted as an HCC-specific marker. The clinical implications have been moderated accordingly.

Reviewer comment 5: Optimal cut-offs were selected using the same dataset without internal validation. Perform bootstrap optimism correction or split-sample validation.

Response: We performed internal bootstrap validation and repeated stratified 10-fold cross-validation. Bootstrap analysis showed an optimism-corrected PIVKA-II AUC of 0.753 versus an apparent AUC of 0.753771, with minimal optimism of 0.000763. However, the bootstrap Youden cutoff showed substantial instability (median 141.78 mAU/mL; 95% percentile range 58.73–715.80). Repeated stratified 10-fold cross-validation produced a mean PIVKA-II AUC of 0.751±0.124, a 95% range of 0.480–0.953, and optimism of +0.002. We chose repeated 10-fold cross-validation rather than a single split-sample analysis because the total sample was modest and a single split would substantially reduce the effective training and testing sample sizes. We now explicitly describe both procedures and their limitations.

Reviewer comment 6: The manuscript should acknowledge symptom burden such as cancer pain and cite PMID 38493063.

Response:We added a brief statement to the Introduction acknowledging the substantial symptom burden of HCC, including cancer-related pain and its impact on quality of life and clinical management, and cited the reference corresponding to PMID 38493063. This addition provides clinical context without changing the primary biomarker research question.

Reviewer comment 7: Specify the manufacturer and model of the automated hematology analyzer and internal/external quality control procedures.

Response: We have added these details. CBC with five-part differential was performed using the Sysmex XN-550 automated hematology analyzer (Sysmex Corporation, Kobe, Japan). Internal quality control used Sysmex XN CHECK control material at appropriate concentration levels, with results monitored against manufacturer-provided targets and acceptable ranges. External analytical quality was assessed through inter-laboratory comparison of selected patient specimens analyzed independently in the study laboratory and a collaborating laboratory. These details are now included in the Laboratory Analysis section.

Reviewer comment 8: For PIVKA-II, report assay lot numbers and external quality control procedures, if available.

Response: We have added the assay lot number and quality-assurance information. PIVKA-II was measured using the Roche Cobas e 411 ECLIA system at AIMS Hyderabad; the assay lot number was 10 08273001. The analytical measurement range was 6.76–12,000 mAU/mL, with values below 40 mAU/mL considered within the manufacturer's normal range. External analytical verification was performed using reference/quality-control samples supplied through an external laboratory, with independent analysis and comparison of results. These details are now reported in the PIVKA-II Quantification section.

Reviewer comment 9: Define early-stage HCC more precisely using BCLC or tumor-size cutoffs and provide the number of patients.

Response: We have clarified the definition. Early-stage HCC is now defined strictly as BCLC-A, consistent with the BCLC staging framework. In this cohort, only 4 of 74 HCC patients (5.4%) were BCLC-A. BCLC-B was classified as intermediate stage. The previously reported BCLC A+B subgroup (n=26) is explicitly identified as early/intermediate rather than early-stage disease and is not equated with BCLC-A. Because only four BCLC-A cases were available, a statistically reliable stage-specific estimate for early HCC could not be derived. This clarification appears in the Methods/Results, Discussion, Limitations, and clinical implications.

Reviewer comment 10: The data availability statements are inconsistent and should be harmonized.

Response: We harmonized the Data Availability Statement throughout the manuscript and submission materials to the following wording: “All relevant data underlying the findings of this study are not deposited in a public repository because of patient privacy considerations and institutional data governance requirements at the University of Sindh, Jamshoro. Anonymised datasets supporting the reported analyses are available from the corresponding author upon reasonable request, subject to a data-sharing agreement with the University of Sindh, Jamshoro.”

Reviewer comment 11: Use a single consistent expansion of PIVKA-II: Protein Induced by Vitamin K Absence or Antagonist-II.

Response: We have standardized the full expansion of PIVKA-II throughout the manuscript to “Protein Induced by Vitamin K Absence or Antagonist-II” and retained the abbreviation PIVKA-II thereafter.

Reviewer comment 12: Use ×10⁹/L consistently instead of interchangeably using ×10³/μL and ×10⁹/L.

Response: We have standardized the platelet and leukocyte concentration units throughout the manuscript and tables to ×10⁹/L, including the CBC tables, ROC analyses, regression tables, and description of derived indices.

Reviewer comment 13: PPV and NPV are derived from the case-control prevalence and may not be generalizable. Explicitly acknowledge this limitation.

Response: We agree and have added an explicit limitation. The study contained 74 HCC cases among 160 participants (46.3%), which is substantially higher than the HCC prevalence expected in many unselected clinical or community screening populations. We now state that PPV and NPV are prevalence-dependent and should not be extrapolated directly to lower-prevalence populations; PPV would generally decrease and NPV increase as prevalence decreases, assuming sensitivity and specificity remain similar. The Clinical Implications section also explicitly states that the observed NPV of 83.9% should not be interpreted as evidence that a low PIVKA-II value can reliably rule out HCC in routine surveillance.

Author-identified correction during revision:

During the revision process, we conducted an additional verification of the ROC analyses for all continuous hematological parameters. This quality-control review identified an inconsistency in the classification direction for platelet count and lymphocyte percentage in the original analysis. Although this issue was not raised by the reviewer, we considered it important to correct it to ensure that the ROC classification direction was consistent with the observed association with HCC.

In the revised analysis, lower platelet counts and lower lymphocyte percentages are correctly represented as the direction associated with HCC discrimination. Platelet count showed an AUC of 0.642 (95% CI: 0.561–0.730), with an optimal cutoff of <148.0 × 10⁹/L, sensitivity of 79.7%, and specificity of 53.5%. Lymphocyte percentage showed an AUC of 0.594 (95% CI: 0.505–0.682), with an optimal cutoff of <23.6%, sensitivity of 74.3%, and specificity of 41.9%.

The correction affects the ROC classification direction and corresponding cutoff interpretation but does not change the underlying AUC estimates. The corrected direction and cutoffs have been updated consistently in the relevant tables, Results, and interpretation. We also re-checked the cross-validation procedure to ensure that the classification direction and Youden-optimal cutoff were determined from the training data only and subsequently applied unchanged to the corresponding held-out test data.

We have made this correction proactively during the revision process to improve the statistical accuracy and internal consistency of the manuscript.

Attachment Submitted filename: Point_to_Point_Response_to_Reviewers_PIVKA_II_updated (1).docx

# Peer review: Decision Letter 1

**DOI:** 10.1371/journal.pone.0361802.r003

**Authors:** Chen Ling

16 Oct 2026

PIVKA-II and Hematological Parameters in Hepatocellular Carcinoma and Non-Malignant Chronic Liver Disease: A Case-Control Biomarker Comparison in a Hepatitis-Endemic Population

PONE-D-26-21745R1

Dear Dr. Hashmi,

We’re pleased to inform you that your manuscript has been judged scientifically suitable for publication and will be formally accepted for publication once it meets all outstanding technical requirements.

Within one week, you’ll receive an e-mail detailing the required amendments. When these have been addressed, you’ll receive a formal acceptance letter and your manuscript will be scheduled for publication.

An invoice will be generated when your article is formally accepted. Please note, if your institution has a publishing partnership with PLOS and your article meets the relevant criteria, all or part of your publication costs will be covered. Please make sure your user information is up-to-date by logging into Editorial Manager at Editorial Manager® and clicking the ‘Update My Information' link at the top of the page. For questions related to billing, please contact billing support.

If your institution or institutions have a press office, please notify them about your upcoming paper to help maximize its impact. If they’ll be preparing press materials, please inform our press team as soon as possible -- no later than 48 hours after receiving the formal acceptance. Your manuscript will remain under strict press embargo until 2 pm Eastern Time on the date of publication. For more information, please contact onepress@plos.org.

Kind regards,

Chen Ling, Ph.D.

Academic Editor

PLOS One

Additional Editor Comments (optional):

Reviewers' comments:

Reviewer's Responses to Questions

**Comments to the Author**

Reviewer #1: All comments have been addressed

Reviewer #2: All comments have been addressed

**********

2. Is the manuscript technically sound, and do the data support the conclusions?

Reviewer #1: Yes

Reviewer #2: Yes

**********

3. Has the statistical analysis been performed appropriately and rigorously?

Reviewer #1: Yes

Reviewer #2: Yes

**********

4. Have the authors made all data underlying the findings in their manuscript fully available?

The PLOS Data policy

Reviewer #1: Yes

Reviewer #2: Yes

**********

5. Is the manuscript presented in an intelligible fashion and written in standard English?

Reviewer #1: Yes

Reviewer #2: Yes

**********

Reviewer #1: The authors have addressed all the comments properly. I have no further comments. I recommend accept.

Reviewer #2: (No Response)

**********

what does this mean?). If published, this will include your full peer review and any attached files.

If you choose “no”, your identity will remain anonymous but your review may still be made public.

**Do you want your identity to be public for this peer review?**  For information about this choice, including consent withdrawal, please see our Privacy Policy

Reviewer #1: No

Reviewer #2: **Yes:**  Wentao Jia

**********

# Editor report: Acceptance letter

**DOI:** 10.1371/journal.pone.0361802.r004

**Authors:** Chen Ling

PONE-D-26-21745R1

PLOS One

Dear Dr. Hashmi,

I'm pleased to inform you that your manuscript has been deemed suitable for publication in PLOS One. Congratulations! Your manuscript is now being handed over to our production team.

At this stage, our production department will prepare your paper for publication. This includes ensuring the following:

* All references, tables, and figures are properly cited

* All relevant supporting information is included in the manuscript submission,

* There are no issues that prevent the paper from being properly typeset

You will receive further instructions from the production team, including instructions on how to review your proof when it is ready. Please keep in mind that we are working through a large volume of accepted articles, so please give us a few days to review your paper and let you know the next and final steps.

Lastly, if your institution or institutions have a press office, please let them know about your upcoming paper now to help maximize its impact. If they'll be preparing press materials, please inform our press team within the next 48 hours. Your manuscript will remain under strict press embargo until 2 pm Eastern Time on the date of publication. For more information, please contact onepress@plos.org.

You will receive an invoice from PLOS for your publication fee after your manuscript has reached the completed accept phase. If you receive an email requesting payment before acceptance or for any other service, this may be a phishing scheme. Learn how to identify phishing emails and protect your accounts at https://explore.plos.org/phishing.

If we can help with anything else, please email us at customercare@plos.org.

Thank you for submitting your work to PLOS One and supporting open access.

Kind regards,

PLOS One Editorial Office Staff

on behalf of

Dr. Chen Ling

Academic Editor

PLOS One
